# T1 relaxation: Chemo-physical fundamentals of magnetic resonance imaging and clinical applications

**DOI:** 10.1186/s13244-024-01744-2

**Published:** 2024-08-09

**Authors:** Michele Gaeta, Karol Galletta, Marco Cavallaro, Enricomaria Mormina, Maria Teresa Cannizzaro, Ludovica Rosa Maria Lanzafame, Tommaso D’Angelo, Alfredo Blandino, Sergio Lucio Vinci, Francesca Granata

**Affiliations:** 1 Radiology Unit - Biomorf Department, University of Messina, Messina, Italy; 2 Neuroradiology Unit – Biomorf Department, University of Messina, Messina, Italy; 3 UOSD Radiology 2 CAST - University Hospital of Catania, Catania, Italy; 4 Department of Radiology and Nuclear Medicine, Erasmus MC, 3015 GD Rotterdam, The Netherlands

**Keywords:** MRI, T1 relaxation time, Magnetism, Chemical shift imaging, Tumbling

## Abstract

**Abstract:**

A knowledge of the complex phenomena that regulate T1 signal on Magnetic Resonance Imaging is essential in clinical practice for a more effective characterization of pathological processes. The authors review the physical basis of T1 Relaxation Time and the fundamental aspects of physics and chemistry that can influence this parameter. The main substances (water, fat, macromolecules, methemoglobin, melanin, Gadolinium, calcium) that influence T1 and the different MRI acquisition techniques that can be applied to enhance their presence in diagnostic images are then evaluated. An extensive case illustration of the different phenomena and techniques in the areas of CNS, abdomino-pelvic, and osteoarticular pathology is also proposed.

**Critical relevance statement:**

T1 relaxation time is strongly influenced by numerous factors related to tissue characteristics and the presence in the context of the lesions of some specific substances. An examination of these phenomena with extensive MRI exemplification is reported.

**Key Points:**

- The purpose of the paper is to illustrate the chemical-physical basis of T1 Relaxation Time.
- MRI methods in accordance with the various clinical indications are listed.
- Several examples of clinical application in abdominopelvic and CNS pathology are reported.

**Graphical Abstract:**

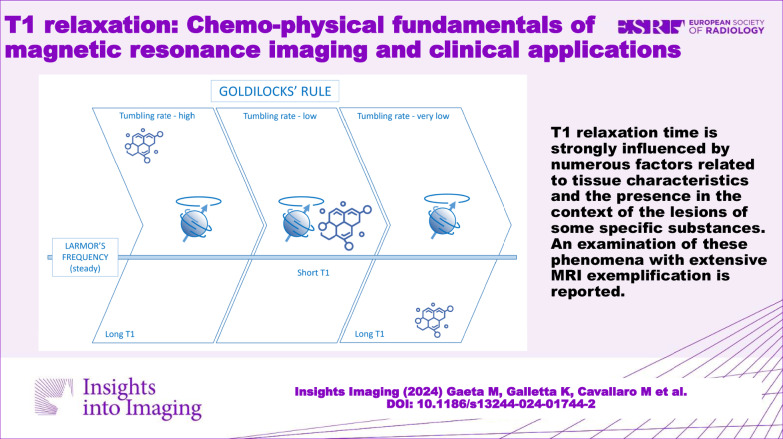

## Introduction

In a very brief overview, MRI could be defined as an anatomic grayscale map of the content of hydrogen atoms (more precisely, of the protons in the nuclei) in the human body and of the interaction of these protons with the surrounding molecular environment.

The core of the MRI signal is the proton density (PD), the quantity of hydrogen protons contained in tissues. No protons, no signal, no MRI. The interaction of protons with surrounding molecular environment determines MRI T1 and T2 relaxation times.

In MRI, electromagnetic energy in the range of radiofrequencies (RF) is absorbed by hydrogen protons that are lifted to a higher energy state. Immediately, the system tries to return to the primitive lower energy condition by a process called relaxation. Two different types of relaxation happen simultaneously as the RF pulse stops: T1 or longitudinal relaxation and T2 or transverse relaxation. T2 relaxation is much quicker than T1.

The bulk of MRI signal in a “standard” spin echo (SE) or gradient echo (GE) magnitude image originates from these three sources: PD, T1, and T2.

Other chemical-physical phenomena like susceptibility, magnetization transfer, and motion (diffusion and perfusion) contribute to the creation of an MRI image. Usually, the percentage of their contribution is small in a classic SE or GE sequence, and it is masked in standard MRI images by the much larger signal dependent from PD, T1, and T2 (the MRI “trimurti”).

These smaller sources of signal can be magnified by well-engineered MRI sequences and can offer important information about normal and pathologic tissues, e.g., diffusion in the early diagnosis of brain ischemia.

The aim of this paper is to illustrate the chemo-physical basis of the T1 relaxation phenomenon, how T1 is influenced, and the way radiologists can use this information in daily practice, applying not conventional MRI sequences. This type of approach allows a kind of “MRI biopsy” of tissues and, consequently, a more effective characterization of pathological processes.

## T1 relaxation time

T1 relaxation time is defined as the time it takes for longitudinal magnetization to reach 63% of the original magnetization. So, T1 expresses the return of protons to the lower energy state on the longitudinal axis, by releasing the absorbed energy to the surrounding environment in the form of heat. Since T1 relaxation is a thermodynamic process, it does not produce an MRI signal.

T1 relaxation properties of a tissue also depend on interactions between protons and the surrounding chemo-physical environment (defined lattice in chemistry applied MR).

Consequently, T1 of a tissue is linked to two main variables: the number of hydrogen protons contained in the tissue and the chemo-physical characteristics of the latex. In other words, T1 relaxation is a useful probe to obtain information on the chemo-physical structure of tissues in the human body.

Every material in the universe, including human tissues, interacts with magnetic fields according to their magnetic susceptibility [1].

Materials can be classified in three fundamental categories: diamagnetic, paramagnetic, and ferromagnetic [2, 3].

Diamagnetic substances, such as fat and water, slightly reduce the strength of the magnetic field. Paramagnetic substances increase the local magnetic field and the signal in T1-weighted MRI images.

The magnetic effect of a substance depends not only on its nature, but also on its concentration. In MRI practice, the effect of paramagnetic substances, and consequently the T1 signal, increases proportionally to its concentration.

## Fundamentals of physics and chemistry explaining T1 relaxation

The factors that primarily influence the T1 signal in MRI are tissue composition, chemical hydrogen bonds, and the possible presence of paramagnetic substances. Another key aspect is the type of MR sequence used.

### Tissue composition—fat

The sources of MRI signal in human body are nearly exclusively the hydrogen protons of water and fat. Since the human body is made up by 70% of water, the main source of signal is water.

On the other hand, in adipose tissue, yellow bone marrow and, to a lesser degree, red bone marrow, skeletal muscle, and liver, all or a part of the signal comes from fat hydrogen.

For Larmor’s Law, the water’s hydrogen protons precess slightly faster than nonpolarized fat molecules. This phenomenon is known as “Chemical Shift”.

In normal condition, the signal from fat is due almost exclusively to triglycerides. Triglycerides are the main energy storage of the human body. They are made up of long chains of CH^2 and are nonpolarized molecules.

The triglyceride peak produces the bulk of fat signal and is considered as the characteristic peak of human fat. However, the MRI spectrum of human fat contains many other peaks, such as cholesterol, phospholipids, and sphingolipids whose contribution to the MRI signal is modest [4].

**Fig. 1:**
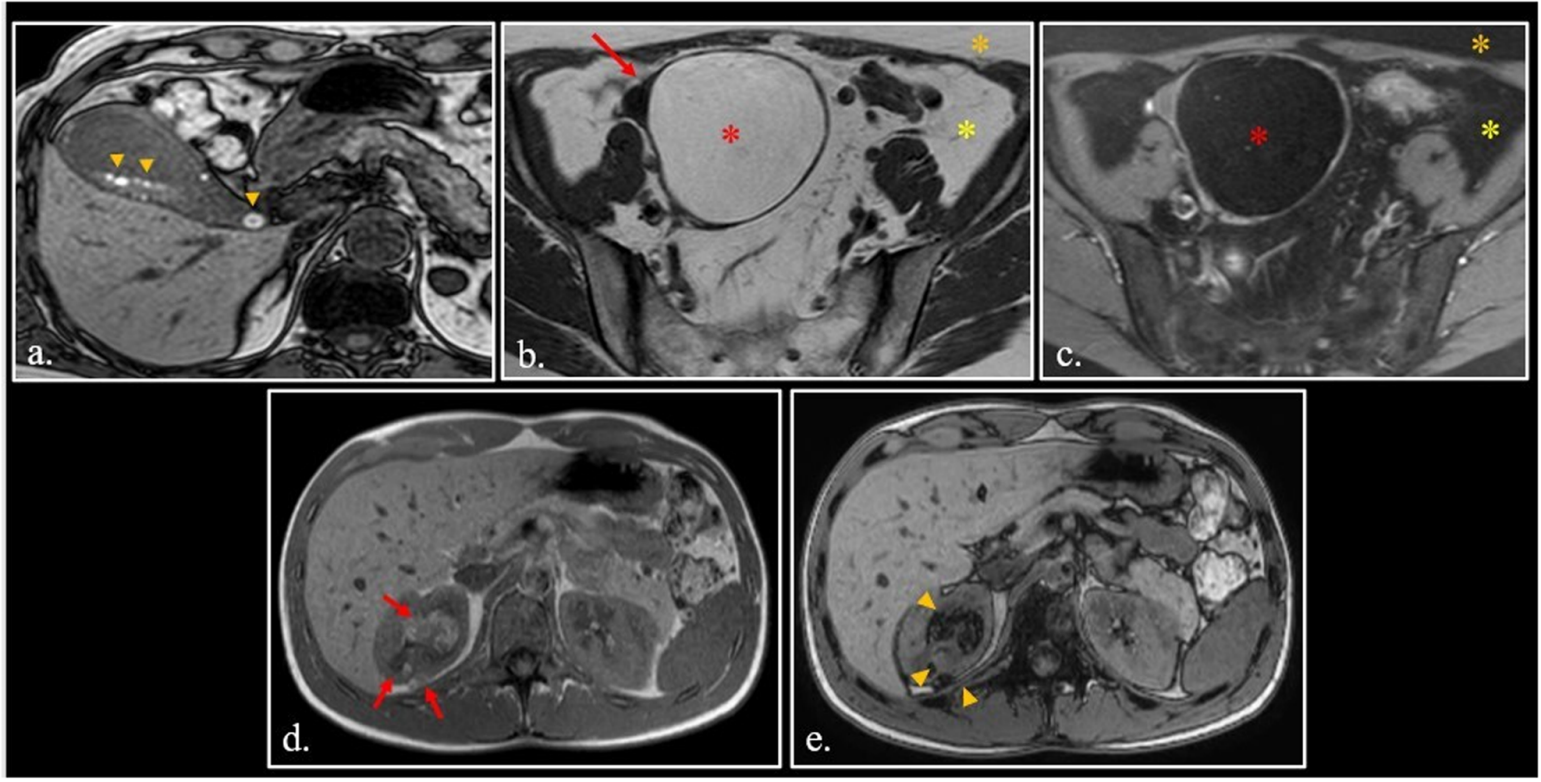
**a** Hyperintense gallbladder stones on GE T1-weighted opposed phase image (arrowheads). **b**, **c** The fat contained in a right ovary dermoid cyst (arrow in (**b**)) is suppressed by a selective chemical shift fat-saturation sequence (**c**), demonstrating that the lipids contained within the cyst (red asterisk) precess at the same rate of triglycerides of the intraabdominal (yellow asterisk) and subcutaneous fat (orange asterisk). **d, e** T1-weighted in-phase (TE 4.6 msec) (**d**)—opposed-phase (2.3 msec) (**e**) scan through the upper abdomen demonstrates multiple angiomyolipomas (arrows) of the right kidney. Since lesions’ voxels contains both fat and water, loss of signal (arrowheads) is seen on the opposed phase image (**e**)

As evidence of this, lesions characterized by high cholesterol content, such as adrenal adenomas, are not hyperintense on T1 scans. Hyperintensity on T1-weighted images of some gallstones or cholesterinic granuloma is not related to cholesterol content but to the presence of a high concentration respectively of glycoproteins and methemoglobin (Fig. 1a).

Lipids contained in some lesions can be different from triglycerides. Ovarian dermoid cysts are a paradigm; since dermoid cysts contains sebaceous glands, the fat we find in these lesions is constituted by sebum that is a complex blend of different lipids. It is composed of triglycerides (≈ 41%), wax esters (≈ 26%), squalene (≈ 12%), and free fatty acids (≈ 16%) [5].

However, the MRI signal of these lipids overlaps with that of triglycerides, and their MRI behavior can be defined “triglyceride-like”. Consequently, their signal is suppressed on selective chemical shift fat-sat sequences (Fig. 1b, c).

### Chemical shift imaging

Coexistence of water and fat in the same voxel can be demonstrated using chemical shift-imaging. Such a possibility has a tremendous value in clinical MRI.

In MRI, not only each proton precesses at Larmor frequency, but also the transverse magnetization vector precesses at the same rate. Consequently, the vectors of water and fat protons at first rotate together (in-phase/IP), then they separate up to stay in opposite positions on the transverse plane (opposed-phase/OP). So, the IP signal intensity is the sum of the two vectors, the OP signal is the result of their subtraction. The phenomenon is cyclic. At 1.5 T the signal is in phase every 4.6 msec, out of phase at 2.3 msec and its multiples.

The simpler chemical shift imaging is based on T1-weighted spoiled dual gradient-echo sequence (Dixon technique) obtaining simultaneously the same image with two different echo-times at 2.3 and 4.6 msec. The coexistence within the same voxel of water and fat causes loss of signal [6, 7]. In voxels containing only fat or only water no change in signal intensity occurs.

**Fig. 2:**
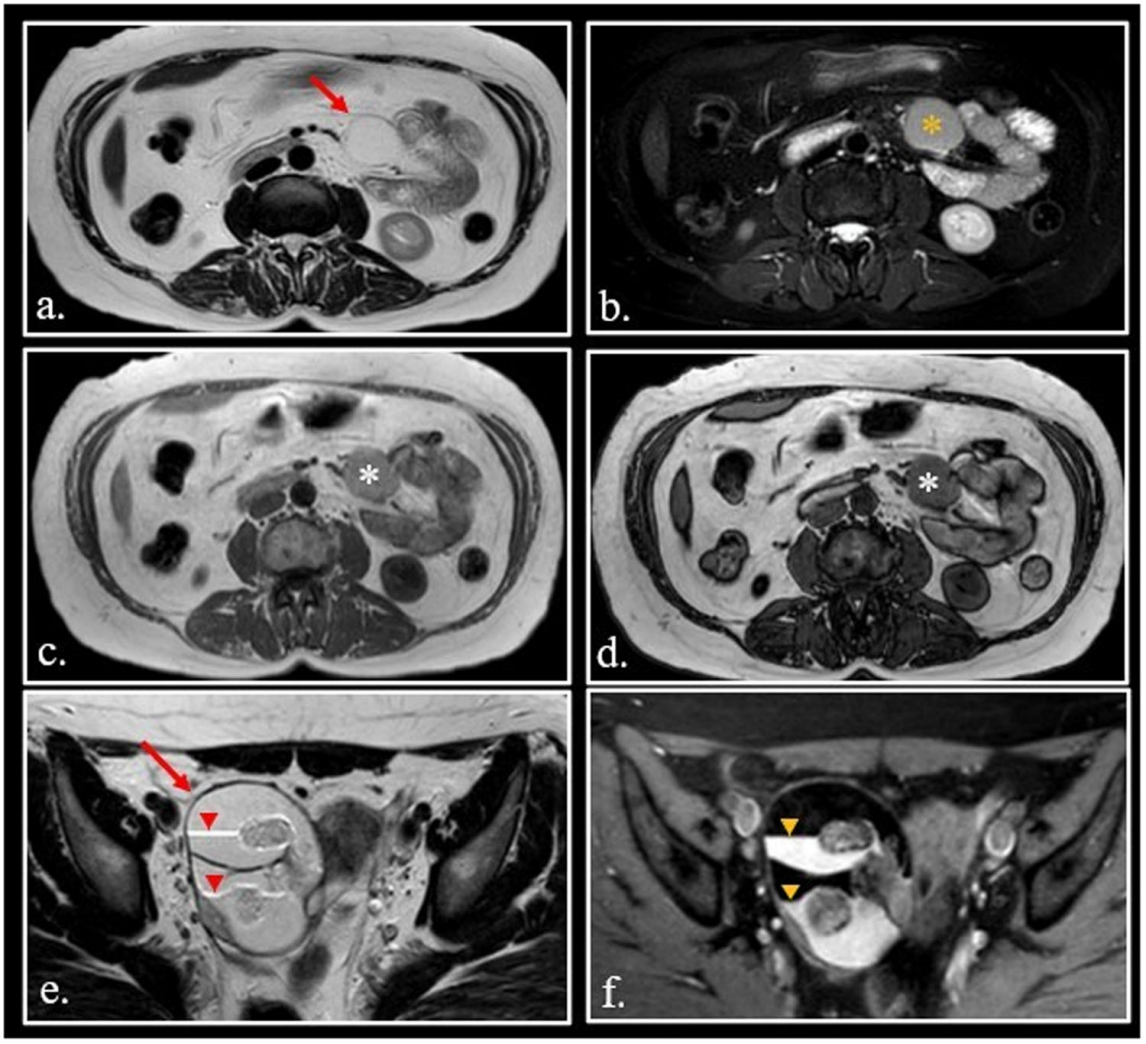
**a–d** Fast Spin-Echo (FSE) T2-weighted scan (**a**) of a 47-year-old female shows a cyst-like lesion of the mesentery root (arrow). FSE T2-weighted fat-sat (**b**) image demonstrates that lesion does not contain pure fat (orange asterisk). On T1-weighted in phase (**c**)—opposed phase (**d**) images loss of signal can be detected (white asterisk). Surgery confirmed the presumptive diagnosis of cystic lymphangioma containing chylous that is a mixed of water and triglycerides. **e**, **f** 25-year-old female with dermoid cyst (arrow). On FSE T2-weighted image (**e**), chemical shift artifact can be seen at the interface between upper fat layer and lower water layer with high-protein content (red arrowheads). On T2-weighted fat-sat (**f**), the chemical shift artifact disappears (orange arrowheads)

Intra-voxel coexistence of fat and water is normal in red bone marrow constituted by 40% of water and 40% of fat, but can also be found in pathological conditions, such as hepatic steatosis, adrenal adenoma, renal angiomyolipoma (Fig. 1d, e), and others. In such cases, the use of chemical shift imaging is a powerful diagnostic tool [8–14] (Fig. 2a–d). In addition, the presence of a chemical shift artifact can be diagnostically useful (Fig. 2e, f).

Finally, by mathematically combining the in-phase and out-of-phase signals, solid water-only fat-suppressed images can be obtained [7].

### Water

In standard MRI, the bulk of the signal originates from the hydrogen of intra and extracellular water.

Three compartments containing hydrogen exist in the tissues.

The first compartment is made by hydrogen contained within macromolecules (e.g., hydroxyl and carboxyl groups) and water tightly bonded to macromolecules (bind water). The relaxation of protons in this compartment is so rapid that MRI signal cannot be measured with standard MRI examinations (so called MRI dark matter).

The second compartment is constituted by water loosely bonded to macromolecules (hydration water). There is a continuous and extremely fast (nanoseconds) exchange of protons between this compartment and the free water.

Free water makes up the third compartment. Free water is the main source of MRI signal.

### Molecular tumbling

The signal intensity in MRI from hydrogen in the different compartments depends on the degree of freedom of the molecules, involved in different chemical bonds, inside tissue environment.

Molecular tumbling is the random movement of rotation, vibration, and translation around the three principal axes of the molecules in the biological environment due to temperature of body. Rotation is the movement that mainly influences the energy exchange between protons and molecules.

The fundamental law is that the energy transfer is faster when the frequency of molecular tumbling is close to the frequency of proton’s precession [15–18].

An excited proton in the vacuum could not release its energy and its T1 relaxation could be as long as months or years--protons need a surrounding environment to relax.

Hydrogen protons that have strong interactions with neighboring molecules will tend to have shorter T1 relaxation times. This can be explained as interactions result in a greater exchange of energy, allowing them to relax faster.

T1 relaxation requires environment fluctuations near the Larmor’s frequency.

The Goldilocks principle takes its name from the 19th-century English fairy tale, “Goldilocks and the Three Bears”, in which a girl named Goldilocks tastes three bowls of porridge and finds she prefers porridge that is neither too hot nor too cold but has just the right temperature to be eaten. The Goldilocks principle is useful in many disciplines, including MRI [19].

In MRI, only the right molecular tumbling rate (not too little, not too much) allows the ideal exchange of energy and shortens T1.

The average rate at which molecules tumble depends on the molecular size. Small molecules (e.g., water) tumble faster than Larmor’s frequency and therefore have long T1 values. The tumbling rate of medium-sized molecules (e.g., lipids) matches well to typical resonant frequencies; therefore, they have short T1 values. On the other hand, macromolecules (e.g., DNA) tumble too slowly to be effective in causing relaxation. So, macromolecules, like small molecules, have long T1 values.

However, when macromolecules accumulate in tissues they reduce the tumbling of the surrounding water, so that the molecular tumbling acquires values that are an average between fast water and slow macromolecules. Consequently, the T1 starts shortening according to the Goldilocks principle (Fig. I Supplementary Materials).

The most common macromolecules that affect MRI signal are glycoproteins.

*Mucin* is largely present in the human body under normal conditions as well as in a lot of pathologic conditions. Normal mucin contains about 95% of water and between 1.5 and 3% of glycoprotein. Due to its high water content, normal mucin has a long T1 and a very long T2. However, when mucin dehydrates, proteins can concentrate up to many g/dL. The raising of protein concentration causes a parallel shortening of T1 until its appears hyperintense on T1-weighted images.

**Fig. 3:**
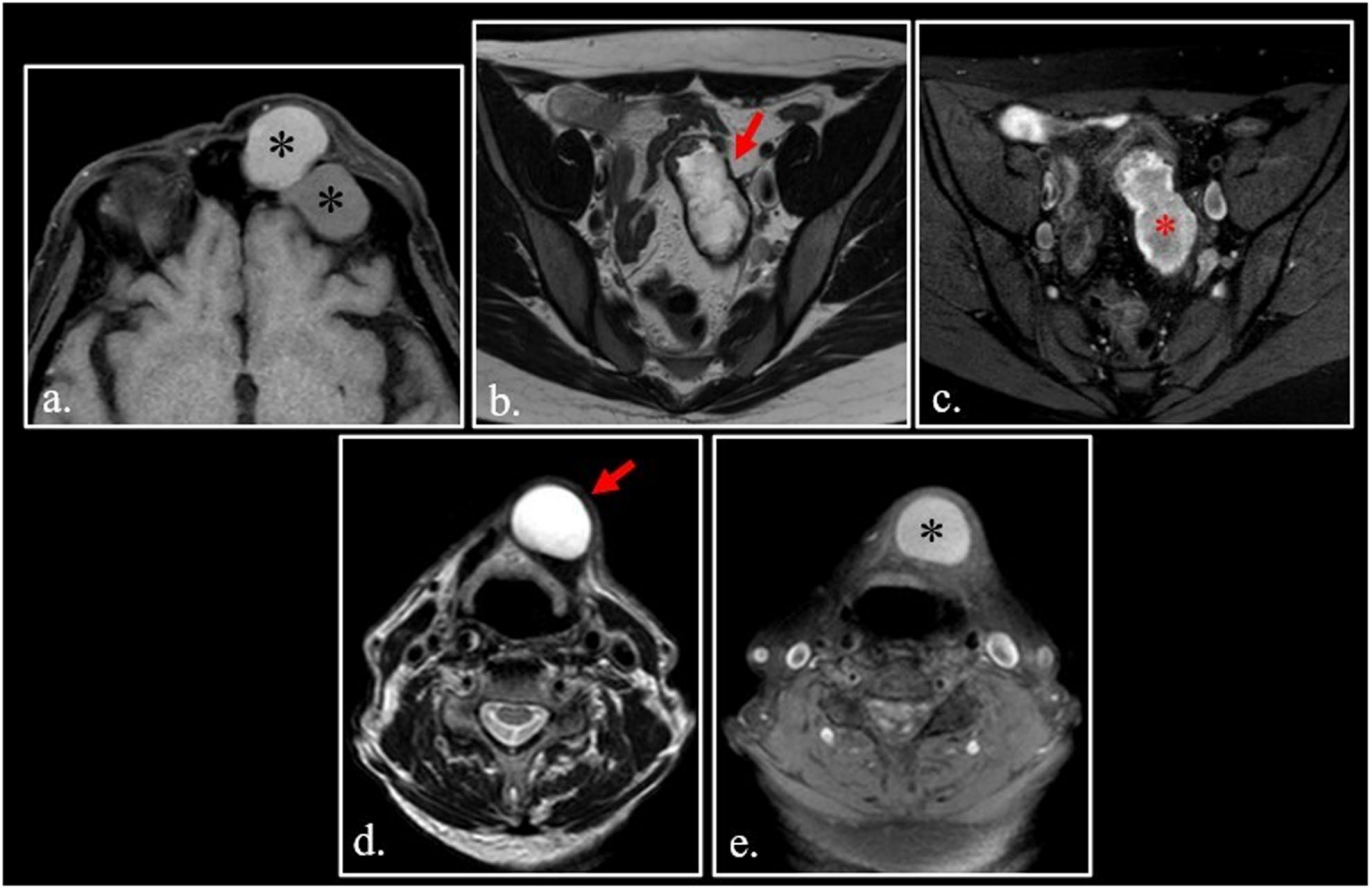
**a** FSE T1-weighted fat-sat scan shows two mucoceles of the left frontal sinus. Different concentration of glycoproteins explains the different hyperintensity of the two lesions (asterisks). **b**, **c** FSE T2-weighted scan (**b**) shows a cystic mucinous borderline cistoadenoma of the left ovary (arrow) with disomogeneous hyperintensity (asterisk) on T1 weighted fat-sat scan (**c**). **d**, **e** FSE T2-weighted (**d**) and T1-weighted fat-sat (**e**) scans of a thyreoglossal cyst (arrow) abutting right strap muscles. T1 hyperintensity (asterisks) correlates to the high content of thyreoglobulin

The list of mucin-containing lesions is long, including both benign and malignant entities [20–22]. Some of these entities can appear hyperintense on T1-weighted images when mucin meets dehydration with a consequent increase of protein concentration (Fig. 3a).

Mucinous malignant tumors (e.g., myxoid liposarcoma, extraskeletal myxoid chondrosarcomas, myxofibrosarcoma, mucinous adenocarcinoma of the ovary) can mimick benign cysts and can show hyperintensity on T1-weighted images [23, 24] (Fig. 3b, c).

*Thyroglobulin* is a glycoprotein containing T3/T4 hormones. High concentration of thyroglobulin can be found in colloid cyst of thyroid, but also in papillary carcinoma and its neck lymph nodes metastases [25, 26].

Thyroglossal duct cysts are the most common congenital cervical anomaly forming anywhere along the thyroid’s route of migration between the tongue base and the inferior neck. They usually develop in the midline in close relation with the hyoid bone. In about 70% of cases, microscopic foci of thyroid epithelium can be found within the cyst wall. Consequently, thyreoglossal duct cysts can contain thyroglobulin and appear hyperintense on T1-weighted images (Fig. 3d, e).

Other macromolecules can influence T1 relaxation time in MRI, and there are several illustrative cases in clinical practice.

**Fig. 4:**
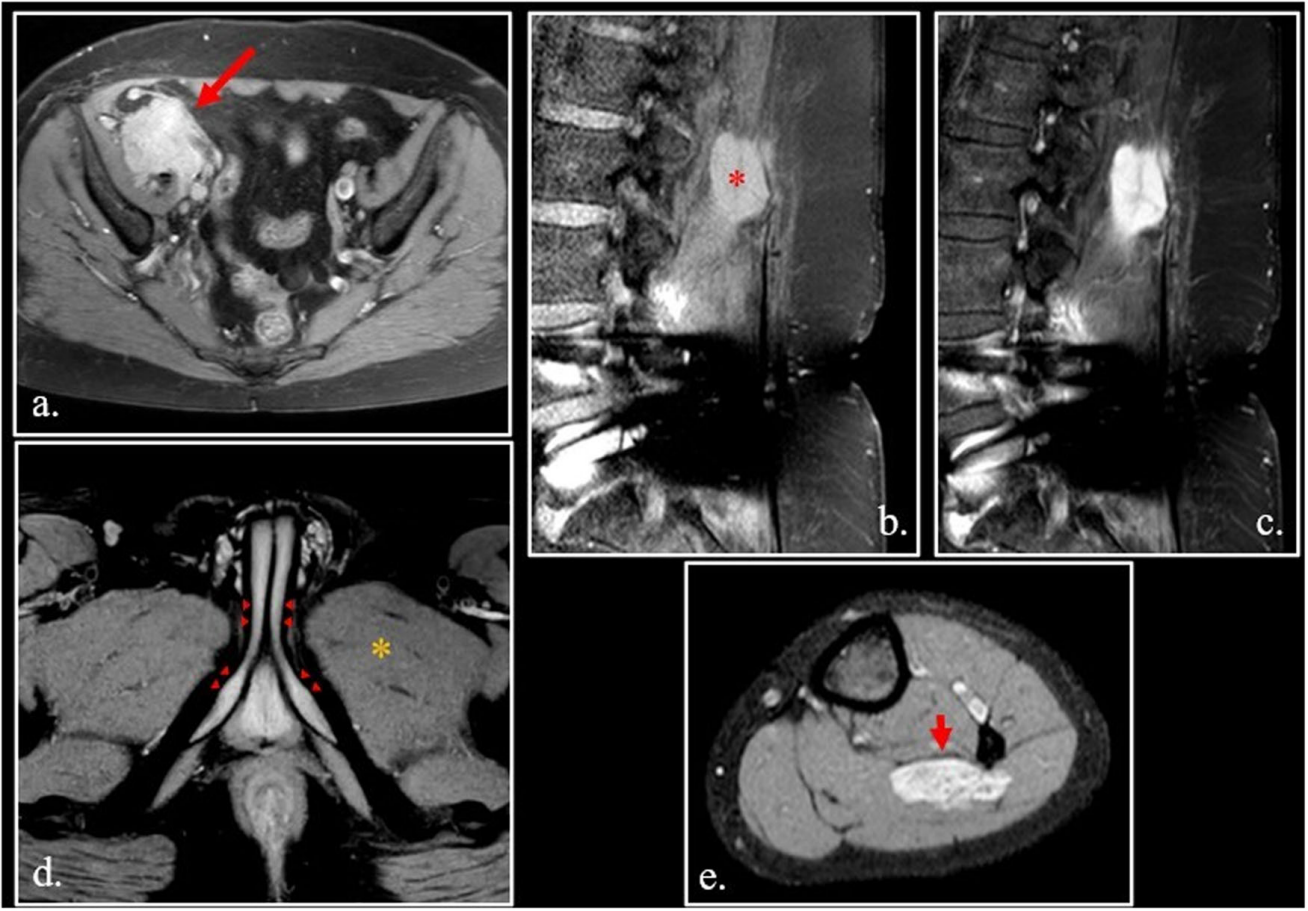
**a** FSE T1-weighted fat-sat of the lower abdomen shows a hyperintense lymphoma (arrow) in the right iliac fossa. **b**, **c** Unenhanced (**b**) and enhanced (**c**) sagittal FSE T1-weighted fat-sat images of hypercellular desmoid of paraspinal muscles (asterisk). The lesion is strongly hyperintense on unenhanced T1-weighted scan due to high cellularity. **d** Unenhanced FSE T1-weighted fat-sat image. The signal intensity of the corpora cavernosa (arrowheads) is quite higher than that of muscles (asterisk). **e** Unenhanced FSE T1-weighted fat-sat image shows a strongly hyperintense hemangioma (arrow) of the soleus muscle

Small round cell cancers like lymphoma can show a slight hyperintensity on T1-weighted images, due to high content of macromolecules in nuclei (including DNA) and the small content of water in cytoplasm (Fig. 4a). For the same reason, some undifferentiated hypercellular desmoid of soft tissue can show T1-hyperintensity [27, 28] (Fig. 4b, c).

Lesions containing a large pool of slowly flowing blood can show hyperintensity on FLAIR and T1 weighted images [29, 30]. Normal corpora cavernosa is the paradigm of this phenomenon in normal conditions, probably related to the high protein content of the blood (Fig. 4d).

Soft tissue hemangiomas can also appear hyperintense to various degrees (Fig. 4e).

**Fig. 5:**
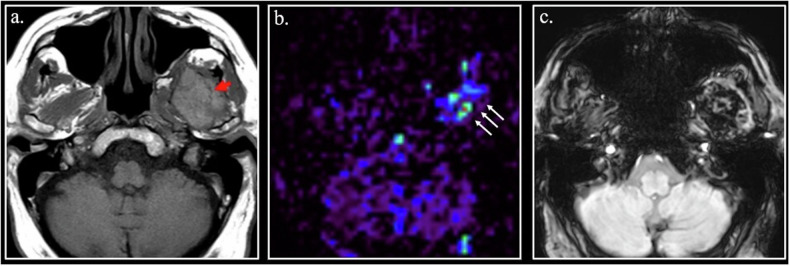
Unenhanced FSE T1-weighted scan (**a**) shows a left masticatory space soft tissue metastasis by clear cell renal adenocarcinoma, resected 10 years before. Intralesional flow-void vessels can be detected into the hypervascularized lesion (red arrow). A pseudo-continuous Arterial Spin Labeling (pCASL) scan (**b**) confirms the high vascularization of the lesions (white arrows). Susceptibility Weighted Imaging (SWI) sequence (**c**) demonstrates intralesional hypointense, slow-flow vessels containing deoxiHemoglobin

Finally, hypervascularized metastases in soft tissues and bones originating from clear cell carcinoma of the kidney can appear hyperintense on fat-sat T1-weighted sequences (Fig. 5a).

Using superb microvascular imaging (SMI) that can display low-speed microvessels [31], it has been demonstrated that renal cell carcinoma is characterized by high-density neo-vascularization with low-speed flow.

Three different causes can explain T1 hyperintensity in clear cell renal cell carcinoma:

1. Presence of microscopic fat [32, 33].
2. Presence of methemoglobin due to diffuse microscopic intra-tumoral hemorrhage.
3. Hypervascularization with high-density intra-tumoral slow-flow microvessels [34, 35] (Fig. 5b, c).

### Gadolinium and other paramagnetic substances

Paramagnetic substances are characterized by unpaired electrons in the outer shell.

Each unpaired spinning electron is a dipole and generates a strong magnetic field (electron dipole).

Dipole-dipole interactions can occur between protons as well as between a proton and an electron. Due to the small size and great gyromagnetic ratio (γ) of an electron, the proton-electron dipolar interaction is much more powerful than a proton-proton interaction.

Paramagnetic substances cause shortening of T1 increasing the local magnetic field and the proton dipole-electron dipole interaction. In such a way, they facilitate the match between stimulated protons and surrounding tumbling molecules.

The paradigm of paramagnetic substances is *Gadolinium* (Gd), the main contrast medium used in clinical MRI. Gd is one of the metals in the Lanthanide series which is strongly paramagnetic due to the presence of 7 unpaired electrons [36, 37].

Other paramagnetic substances can accumulate in human tissues due to pathologic processes such as: methemoglobin (Met-Hb), melanin, hydroxyl radicals (·OH), manganese (Mn), cupric ions (CU_2 + _), molecular oxygen (O_2_), and some metals contained in medical devices.

*Methemoglobin* (met-Hb) is the product of changes of hemoglobin after intra-tissue hemorrhage. Although met-Hb is a macromolecule, its main effect on T1 is because it owns a ferric ion (Fe_3 + _) containing five unpaired electrons. In addition, its spatial structure allows a close approach of water molecules to the ferric ion (so called inner sphere relaxation). Consequently, met-Hb is highly paramagnetic and its presence results in very short T1 values [38].

During the first week after hemorrhage, met-Hb is compartmentalized into cells. This compartmentalization generates magnetic susceptibility effects, so that hematoma appears dark on T2/T2*-weighted images but does not influence T1 signal. After lysis of red blood cells, met-Hb is released into the extracellular spaces with disappearance of T2* dephasing effects [39].

**Fig. 6:**
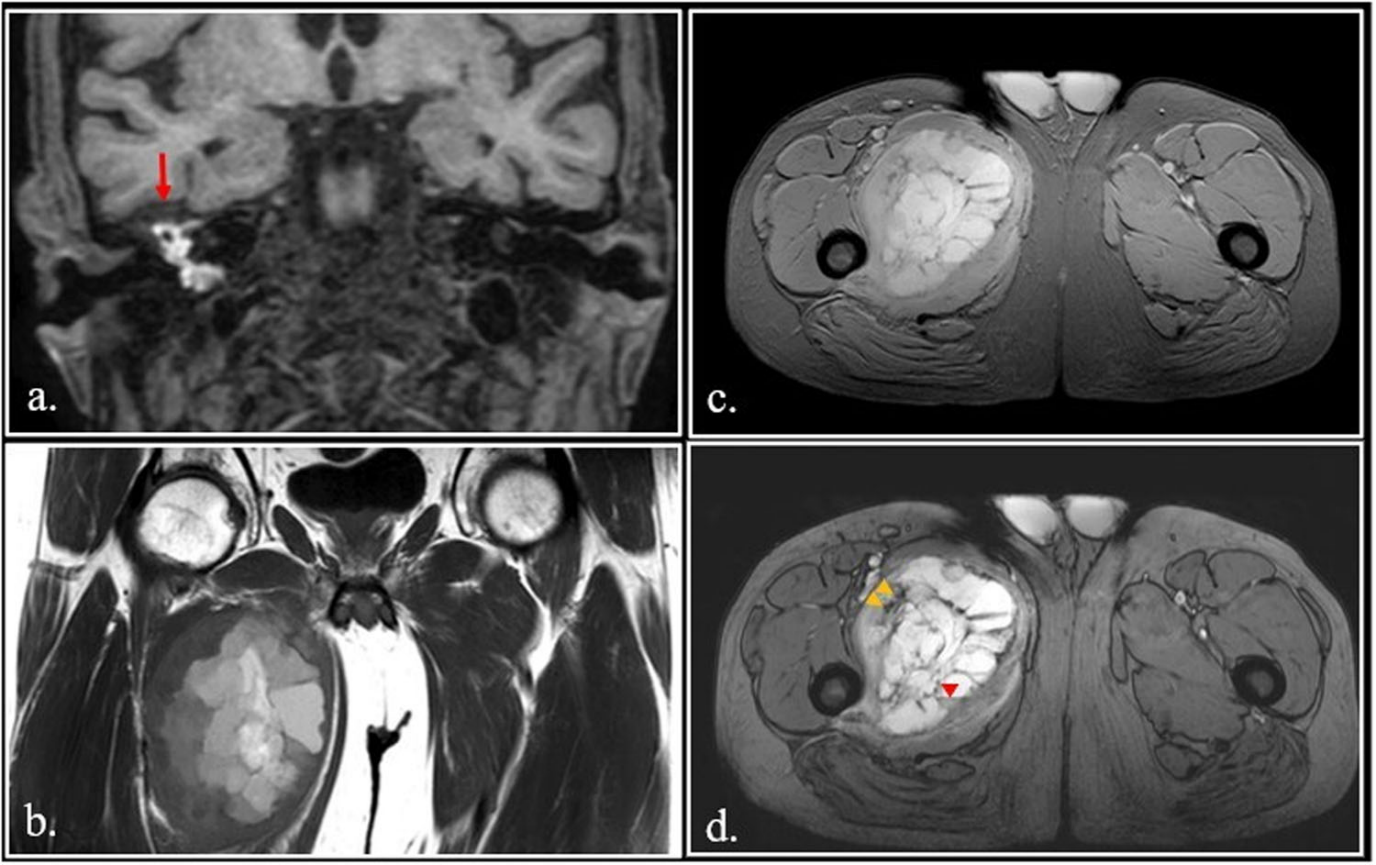
**a** Coronal FSE fat-sat T1-weighted image. Typical high signal cholesterol granuloma (arrow) of the right middle ear. **b–d** Multicystic telangiectatic soft tissue sarcoma of the abductor muscles of the left thigh. On Coronal FSE T1-weighted image (**b**), the cysts demonstrate different degree of hyperintensity due different concentration of T1 shortening substances. Dual Gradient-Echo (GE) T2*-weighted images obtained at 20 ms (**c**) and 40 ms (**d**) show fluid-fluid interfaces. At 40 ms (**d**), there is a blooming by susceptibility artifact at the fluid-fluid interfaces (red arrowhead) and in the solid component of the neoplasm (yellow arrowheads). The susceptibility phenomenon demonstrates that the hyperintensity of the lesion is due to methemoglobin in a hemorrhagic lesion

In some cases, the presence of met-Hb can be a very useful finding to obtain a high-confident diagnosis, as in cholesterol granuloma of the middle ear and petrous apex (Fig. 6a). In other cases, met-Hb content in cystic soft tissue sarcomas can be a diagnostic challenge in differentiating hematomas from neoplasms [40] (Fig. 6b–d).

The demonstration of paramagnetic substances using sequences sensitive to susceptibility allows for the differentiation of T1-hyperintense lesions containing mucin from lesions containing methemoglobin.

*Melanin* is a broad term, which refers to a group of pigments constituted by high-weight molecules. Melanin is normally contained in human skin and brain (neuromelanin) [41].

Melanin is paramagnetic. Its paramagnetism is not due to its proteinic nature, but to the presence of paramagnetic metals within its molecule [42].

**Fig. 7:**
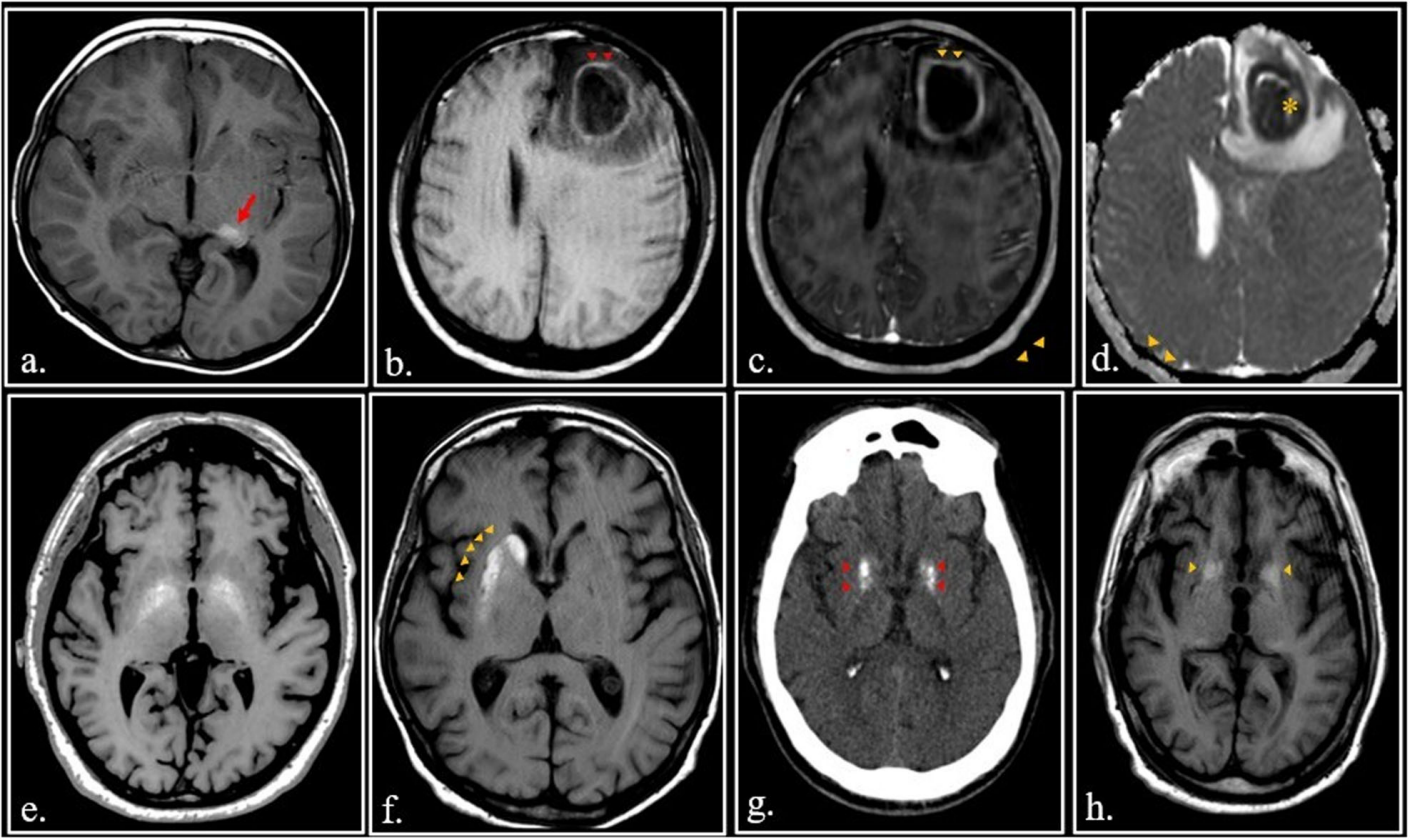
**a** 7-year-old girl with leptomeningeal and intraparenchymal melanosis. Axial SE T1-weighted image shows an hyperintense melanin-containing nodule in the left posterior hippocampus (arrow) **(b–d**) A high signal ring (red arrowheads) can be seen on unenhanced FSE T1- weighted scan in a left frontal brain abscess (**a**) with intense enhancement (yellow arrowheads) after Gadolinium administration (**b**). Apparent Diffusion Coefficient (ADC) map (**c**) shows the typical restriction (asterisk) of the abscess. **e** 62-year-old man with hepatic failure and parkinsonism. FSE T1-weighted image shows bilateral hyperintensity of globus pallidus (arrowheads). **f** 58-year-old diabetic female with a rapid onset of hemichorea-hemiballismus. Marked hyperintensity of the right putamen and caudate nuclei (arrowheads) is visible on unenhanced FSE T1-weigthed image. **g**, **h** 64 years-old psychiatric patient with Fahr disease. CT scan (**a**) shows bilateral calcification of the globus pallidus (red arrowheads). On FSE T1-weighted image (**b**), the calcification appears hyperintense (orange arrowheads)

Melanotic melanoma can appear hyperintense on T1-weighted images (Fig. 7a).

Soft tissue clear cell sarcoma contains melanin and can show high signal on T1-weighted images, mimicking a soft tissue metastasis from melanoma [43].

Macrophages contain *hydroxyl radicals*, which are also paramagnetic. In the wall of abscesses innumerable macrophages can be found; thus, the abscess wall shows a characteristic high signal intensity on unenhanced T1- images [1] (Fig. 7b–d).

*Manganese* is strongly paramagnetic because its electronic shells contain five unpaired electrons. For this reason, Mn-based intravenous contrast medium is considered an alternative to Gd for MRI [44, 45].

Traces of Mn are normally contained in the human body functioning as a cofactor for a variety of enzymes. Hepatic encephalopathy can develop in patients with hepatic failure and is caused by the accumulation of neurotoxins, including manganese, in the globus pallidus and substantia nigra, with a characteristic increase of signal intensity in the basal ganglia on T1-weighted images [46–48] (Fig. 7e).

Accumulation of manganese in the basal ganglia can also occur in hyperalimentation or long-term parenteral nutrition and in hereditary hemorrhagic telangiectasia [49].

Non-ketotic hyperglycemia can cause hemichorea-hemiballismus syndrome in which the involvement of putamen and subthalamic nuclei generates a movement disorder (Fig. 7f). On T1-weighted images, the basal ganglia appear hyperintense. It is now hypothesized that hyperintensity is related to manganese accumulation [50–52].

*Copper* is diamagnetic. On the other hand, its ion, Cu^2+^, is paramagnetic.

In a cirrhotic liver, regenerative nodules, dysplastic nodules, and HCC can contain Cu^2+^ [53] and show hyperintensity on unenhanced T1-weighted images [54–56]. Thus, hyperintensity on T1-weighted images does not allow a differential diagnosis between benign and malignant hepatic nodules.

In addition, other causes of hyperintensity of cirrhotic nodules on T1-weighted images exist, due to the presence of other substances, such as glycogen, fat, and highly concentrated proteins [57, 58].

It is worthy to note that *Calcium* is weakly paramagnetic due to the so-called Pauli’s paramagnetism [59, 60]. The chemical composition of pathological and physiological calcification in human body is complex including crystalline Ca_3_(PO_4_)_2_, hydroxylapatite, and a miniscule amount of copper (Cu), manganese (Mn), zinc (Zn), magnesium (Mg), iron (Fe), mucopolysaccharides, and proteins binding the mineral ions, in addition to calcium [61].

Consequently, MR signal of these calcifications may vary with potentially high-signal intensity on T1-weighted images, such as in Fahr disease [62] (Fig. 7g, h).

### Rescaling

T1 shortening can make a tissue hyperintense. We can define hyperintensity only qualitatively. Every tissue with a signal on T1-weighted images higher compared to the signal of normal surrounding tissues can be defined as hyperintense.

Scaling is a linear transformation that changes the size of a mathematical object [63].

**Fig. 8:**
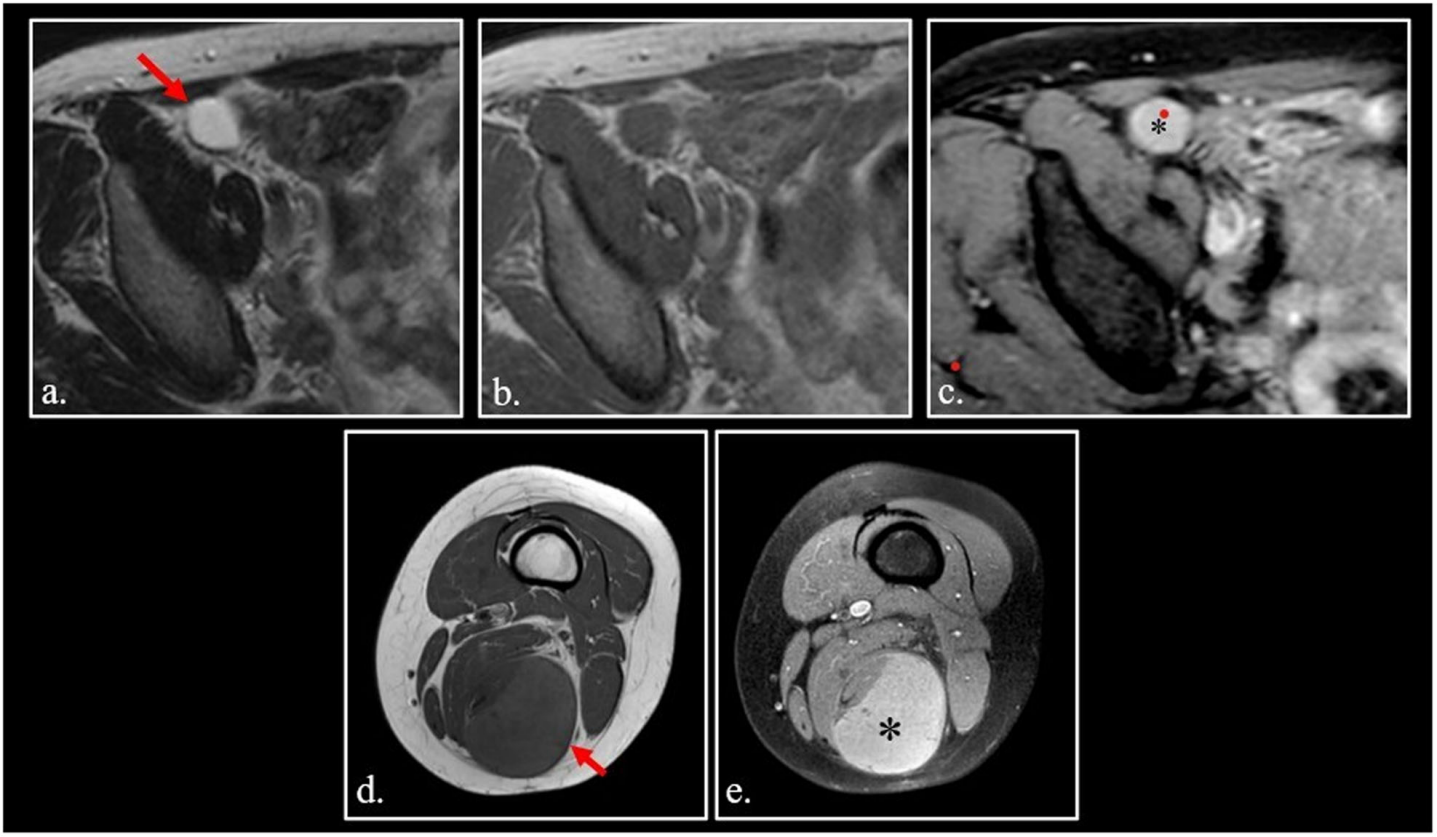
**a–c** Appendicular mucocele showed on FSE T2-weighted (**a**), T1-weighted (**b**) and T1-weighted fat-sat (**c**) scans. Note the rescaling phenomenon on T1-weighted fat-sat scan (asterisk). **d**, **e** Unenhanced FSE T1-weighted (**d**) and T1-weighted fat-sat (**e**) of a 35-year-old female with rapidly growing clear cell sarcoma of the left thigh (arrow). Note the rescaling phenomenon in (**e**) (asterisk)

Scaling of radiological interest usually involves the image matrix. However, another important scaling exists in MRI and occurs in T1 fat-sat sequence. Sometimes, tissues that show an intermediate signal intensity on T1 weighted images can appear hyperintense on T1 fat-sat images. Such a phenomenon is known as rescaling [64, 65] (Fig. 8).

In MRI, the numerical value of each voxel’s signal is displayed on a fixed grayscale range, so the signal intensity is not absolute but relative to that of other voxels. On T1 fat-sat, the fat signal that occupies the highest value of the range is eliminated, consequently the intensity of other voxels is redistributed (rescaled) to fill the values left empty from nulled fat.

Such a phenomenon can result in higher signal intensity of a tissue on T1 fat-sat sequence in comparison with non-fat-sat T1 and can simulate enhancement (pseudo-enhancement from rescaling).

From the practical point of view, such a misinterpretation can originate from the comparison of T1 non-fat-sat non-contrast-enhanced images with T1 fat-sat contrast-enhanced images. Misdiagnosis can be easily avoided comparing or subtracting pre- and post-enhanced T1 fat-sat images (Fig. II-III Supplementary Materials).

### T1 effect on T2. The “shading sign”

A common statement is that T1 and T2 relaxations are MRI phenomena that occur simultaneously and independently. However, T1 and T2 are not completely independent. The influence of T1 on T2 is due to the fact that during the rotation of the magnetization vector on the transversal plane, protons start returning on the longitudinal plane. In this way, T1 relaxation subtracts protons to transverse magnetization vector. In normal conditions, T1 is too long to significantly influence T2 relaxation.

When T1 is very shortened, the rapid shift of protons along the longitudinal axis strongly reduces the magnitude of the transverse magnetization vector and causes weakening of the MRI signal. Such a phenomenon can cause lowering, up to complete disappearance, of the MRI signal on T2-weighted images when T1 is almost equal or shorter than the echo time (TE) of T2 sequence.

This finding is known as *shading sign* and has been initially considered typical for ovarian endometrial cyst. Short T1 of endometrial cysts is explained by the high content of protein and iron from repeated bleeding [66, 67].

However, the shading sign is not specific and can be found in every pathologic process with a sufficiently short T1 due to either high protein content or the presence of paramagnetic substances [68, 69].

**Fig. 9:**
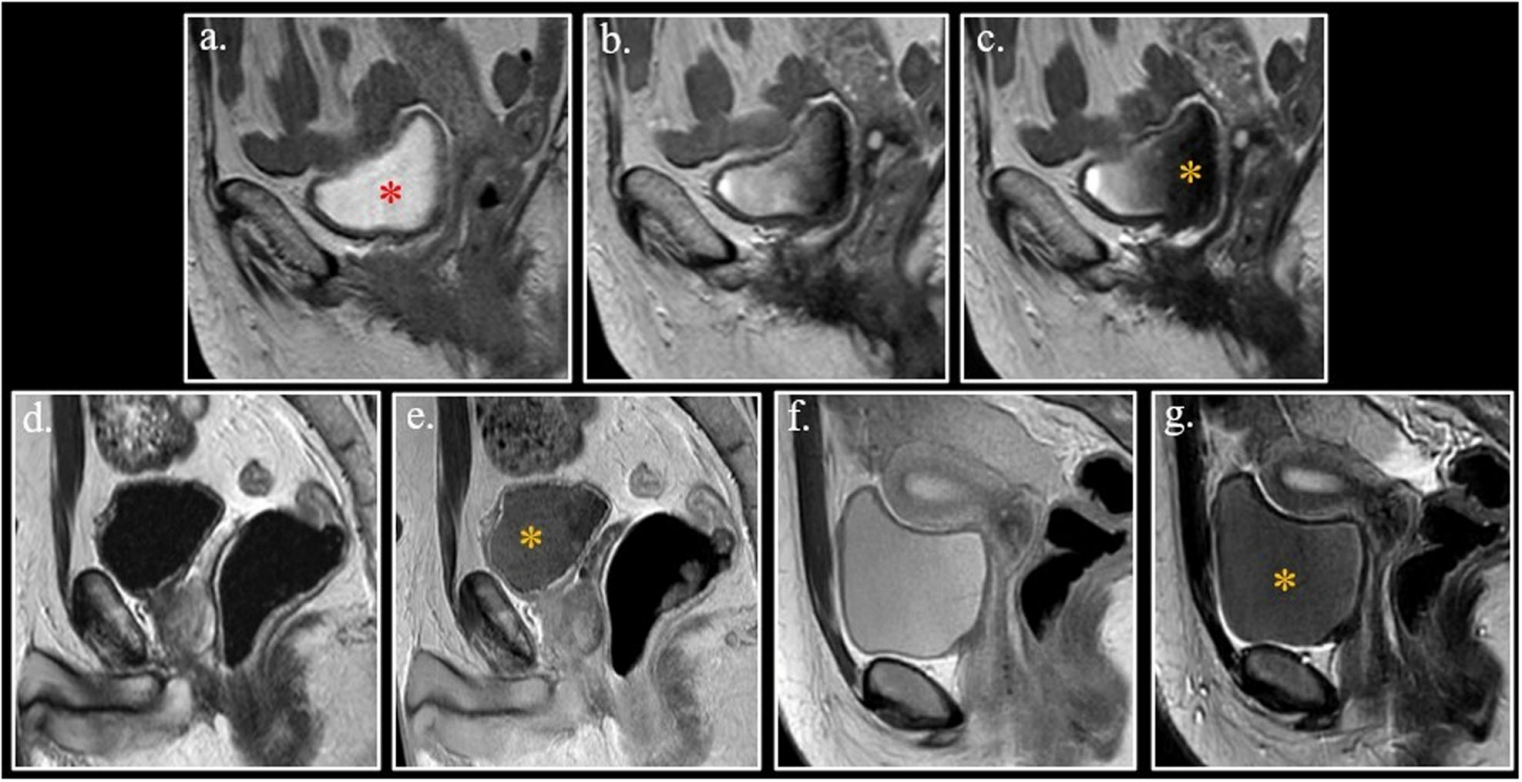
**a**–**c** On FSE T1-weighted sagittal image (**a**) of the bladder, homogeneous high signal of urine can be seen (red asterisk). A FSE T2-weighted image obtained immediately after T1 sequence shows a layering of Gadolinium. A lower strip of urine with more concentrated Gadolinium defines the so-called shading sign (**b**). After 15 minutes (**c**), the further concentration of Gadolinium causes enlargement of the shading sign (orange asterisk). **d**, **e** “Disappearance” of the urine signal on T2-weighted image due to diffuse high concentration of Gadolinium in the bladder. Sagittal T2-weighted (**d**) and T1-weighted images (**e**) after Gadolinium administration. The high concentration of Gadolinium causes the shading phenomenon on T2-weigthed image (**d**). In addition, there is a loss of signal of the urine also on T1-weighted image (yellow asterisk in (**e**)) because in this case the T2* of urine is longer than TE of T1 sequence, preventing the collection of MR signal. **f, g** A sagittal FSE double echo images at 40 ms (**f**) and 100 ms (**g**) through the bladder demonstrated the influence of different echo-times on the appearance of the shading sign. The shading sign (asterisk) can be seen only at TE 100 ms (**g**)

The raising concentration of Gd in the bladder is a good example of different effects on T2 due to the progressive T1 shortening of urine signal (Fig. 9a–c).

At the same time, when T2 is strongly reduced and its value is inferior to the TE of T1 sequence, the signal of T1 cannot be collected either, due to the rapid disappearance of the free induction decay (FID) (Fig. 9d, e).

It is noteworthy that the use of different echo-times can strongly affect the intensity of the shading phenomenon (Fig. 9f, g).

**Fig. 10:**
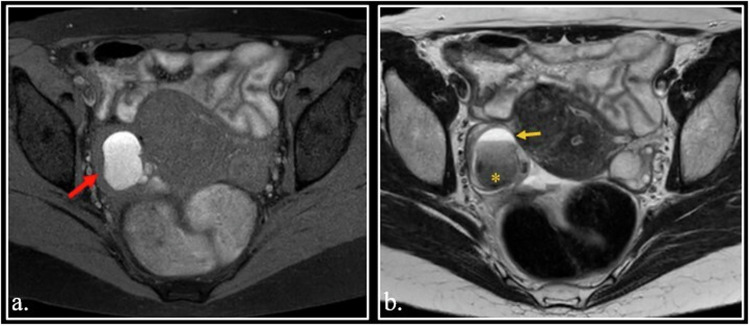
Axial FSE unenhanced T1-weighted fat-sat image (**a**) shows a homogeneously hyperintense endometrioma (red arrow) of the right ovary. On Axial FSE T2-weighted image (**b**), the lesion is hypointense (asterisk) (shading sign) with a thin layer of hyperintensity in the upper part of the cyst (orange arrow) due to lower concentration of T1-shortening substances

The most common pathology showing the shading sign is endometriosis. Accumulation of both blood proteins and hemoglobin degradation products cooperate in creating the typical MRI appearance of endometriosis cysts (endometriomas). (Fig. 10–Fig. IV-VI Supplementary Materials).

### “T2 blackout” effect

T2 blackout effect is a phenomenon that can cause diagnostic confusion in diffusion-weighted imaging. It represents the reverse of T2 shine through [70].

Since EPI diffusion images are T2* weighted, lesions with a short T2 can affect the diffusion sequence. Every cause of T2 shortening, including a very short T1, can produce a false reduction of the values on the ADC map.

Consequently, ADC measurement of a lesion strongly hyperintense on T1 is not reliable (Fig. VII Supplementary Materials).

The presence of low signal intensity on b images is the clue to avoid misdiagnosis.

## Nontypical T1-weighted sequences

### Susceptibility weighted imaging

It is underrecognized that SWI sequence does not depend exclusively on T2* relaxation. SWI is a steady state sequence and, although T2* and phase effects dominate, the signal intensity is also a function of T1 [71, 72].

Consequently, T1 contrast-weighting can appear both on noncontrast-enhanced sequences and after Gd administration. This phenomenon is known as “T1 shine-through effect” [73, 74]. The use of a shorter TE of 10 ms, and a flip angle larger than 20° can increase the “T1 shine-through effect” [75] (Fig. VIII Supplementary Materials).

**Fig. 11:**
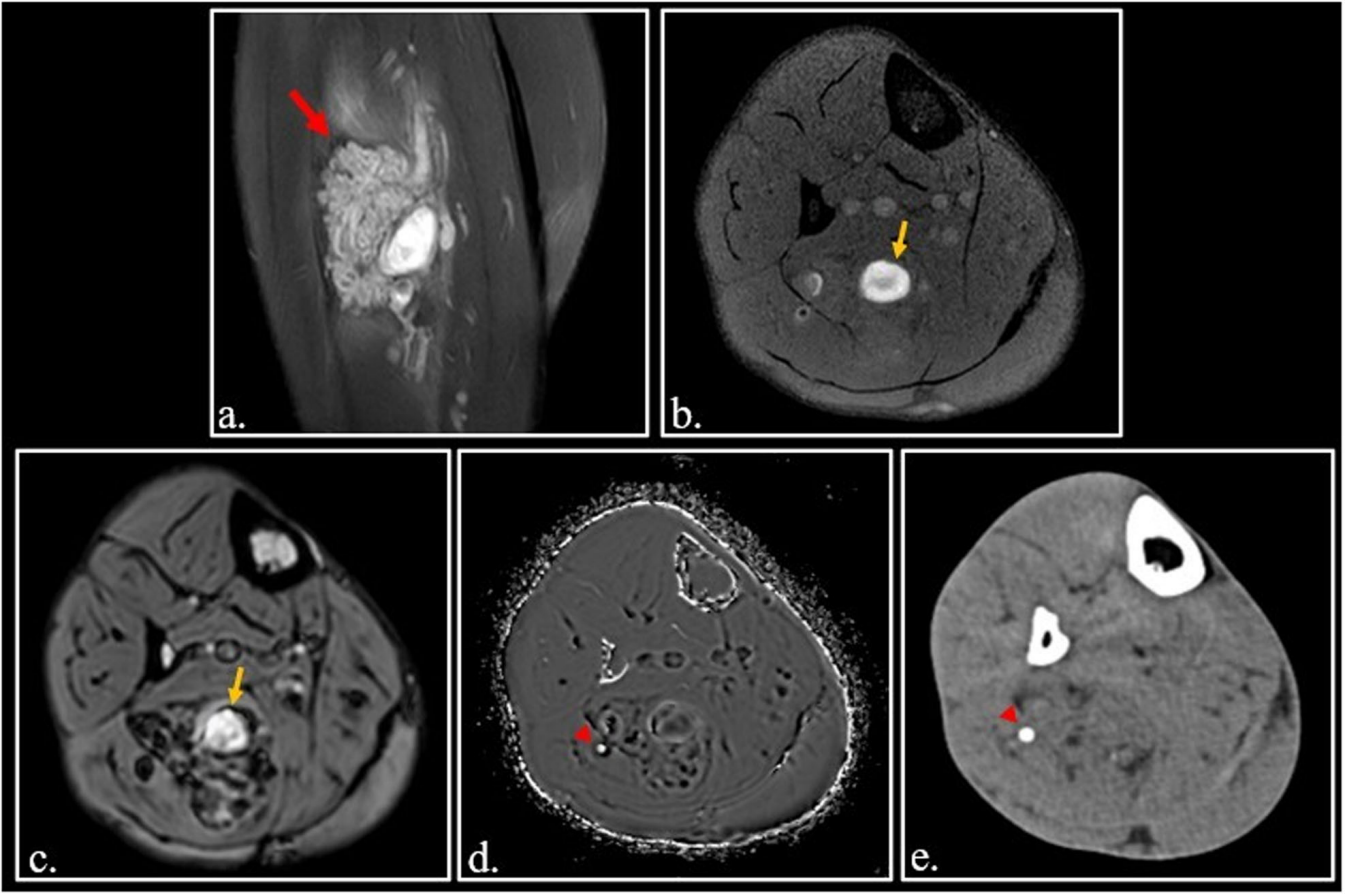
Sagittal FSE PD-weighted fat-sat (**a**) and Axial FSE T1-weighted (**b**) images in a 25-year-old male with muscular slow-flow malformation of the right calf (red arrow in (**a**)). An intralesional hyperintense hematoma is visible on the T1-weighted image (orange arrowhead). On SWI (**c**), the hematoma appears hyperintense (orange arrow). It is surrounded by multiple hypointense spots due to intracellular deoxyhemoglobin into vessels. On phase map (**d**), a white spot due to a calcified phlebolith (arrowhead) can be detected at the margin of the lesion. CT scan examination (**e**) confirms the presence of a calcified phlebolith (arrowhead)

Although SWI sequence is usually used for scanning brain, its value outside the brain should be better evaluated (Fig. IX Supplementary Materials - Fig. 11).

### Steady-state sequences

Balanced steady-state is a fast GE sequence in which the image contrast depends upon the T2- to T1-ratio (T2/T1). Steady-state sequences are both fast and robust with high SNRs and contrast-to-noise ratios and are commonly used in MR imaging. They are the standard for cine cardiac imaging and are also widely used in abdominal imaging, cardiac imaging and neuroimaging.

Commercial names for balanced steady-state sequence include TrueFISP (Siemens), FIESTA (GE), Balanced-FFE (Philips), BASG, or True SS.

The balanced steady-state images are primarily T2-weighted and are commonly clinically used for this type of weighting. However, the component of T1-weighting explains why fat and lesions with short T1 appear hyperintense on this sequence. In addition, steady-state sequences are sensitive to Gd which reduces T1 and consequently increases T2/T1 ratio [76–79] (Fig. X-XI Supplementary Materials).

### Inversion recovery (IR) sequences

In IR sequence, a 180° radiofrequency pulse is used before a turbo spin echo sequence.

The time interval between 180° and 90° pulses is known as inversion time (IT). IR sequences allow to selectively null signal from certain tissue using an appropriate IT (called IT null) which can be calculated with the simple equation: T1 of the tissue we want to suppress multiplied natural logarithm of 2 (value 0.69) (log TInull = T1 • (ln 2) ≈ 0.69 x T1).

The two fundamental IR sequences are STIR, using IT of 170 msec to null the fat signal, and FLAIR, which uses IT of 2000 ms to suppress liquor signal.

IR sequences intrinsically have a double weighting. Conventional IR are T2-weighted, but the use of inversion time introduces a simultaneous T1-weighting since every abnormality changing the T1 of a tissue does not permit to null its signal. This unsuccessful suppression is paradoxically a useful diagnostic finding [1]. This phenomenon explains why contrast-enhanced FLAIR is the most sensitive sequence in diagnosing meningitis. The passage of a small amount of Gd through inflamed meninges is not detected by T1-weighted sequences but changes enough the T1 of liquor to prevent the suppression of its signal in FLAIR images, consequently appearing hyperintense [80–82] (Fig. XII Supplementary Materials).

Short and medium-TI sequences, including STIR, have an additional T1 weighting due their additive T1 plus T2 contrast [83]. On the other hand, in SE and TSE sequences lesions usually have prolonged T1 and T2 with competitive T2 less T1 effects on signal.

A typical example of the advantage of STIR on T2 TSE sequence is in the detection of spinal cord demyelinating plaques (Fig. XIII Supplementary Materials).

However, STIR has a major drawback: fat suppression is not selective. Since STIR suppresses the signal based on T1, every lesion with a short T1 like that of fat is nulled, potentially causing misdiagnosis [1, 84, 85] (Fig. XIV Supplementary Materials).

For the same reason, STIR must not be used after Gadolinium administration because the signal from contrast-enhanced tissues will also be nulled [1].

## T1 mapping

In recent years, technological innovation has led to the development of new techniques that allow direct quantification of T1, extracellular volume fraction (ECV) and T2 values on an absolute scale.

Although the modifications of these values are not specific, they allow to evaluate the alterations in tissue composition through quantitative parametric maps.

T1 mapping exploits acquisition of different T1 weighting along the longitudinal recovery curve, which are used to generate color-coded maps [86]. Several approaches have been proposed for the evaluation of T1 mapping, the most widely used are IR sequences, such as multiple single-shot bSSFP acquisitions, including MOdified Look Locker Inversion recovery (MOLLI) and shortened MOLLI (ShMOLLI) [87–89].

T1 mapping of the myocardium is the epitome of the clinical use of this method. Native T1 mapping allows the identification of alterations in the myocardial tissue that could be indicative of the presence of an underlying pathology without the need to administer a contrast medium. Many pathologies can be responsible for alterations in T1 values, including diffuse myocardial fibrosis, edema, inflammation, and infiltrative diseases (amyloidosis, Fabry disease, hemosiderosis) [89–91].

**Fig. 12:**
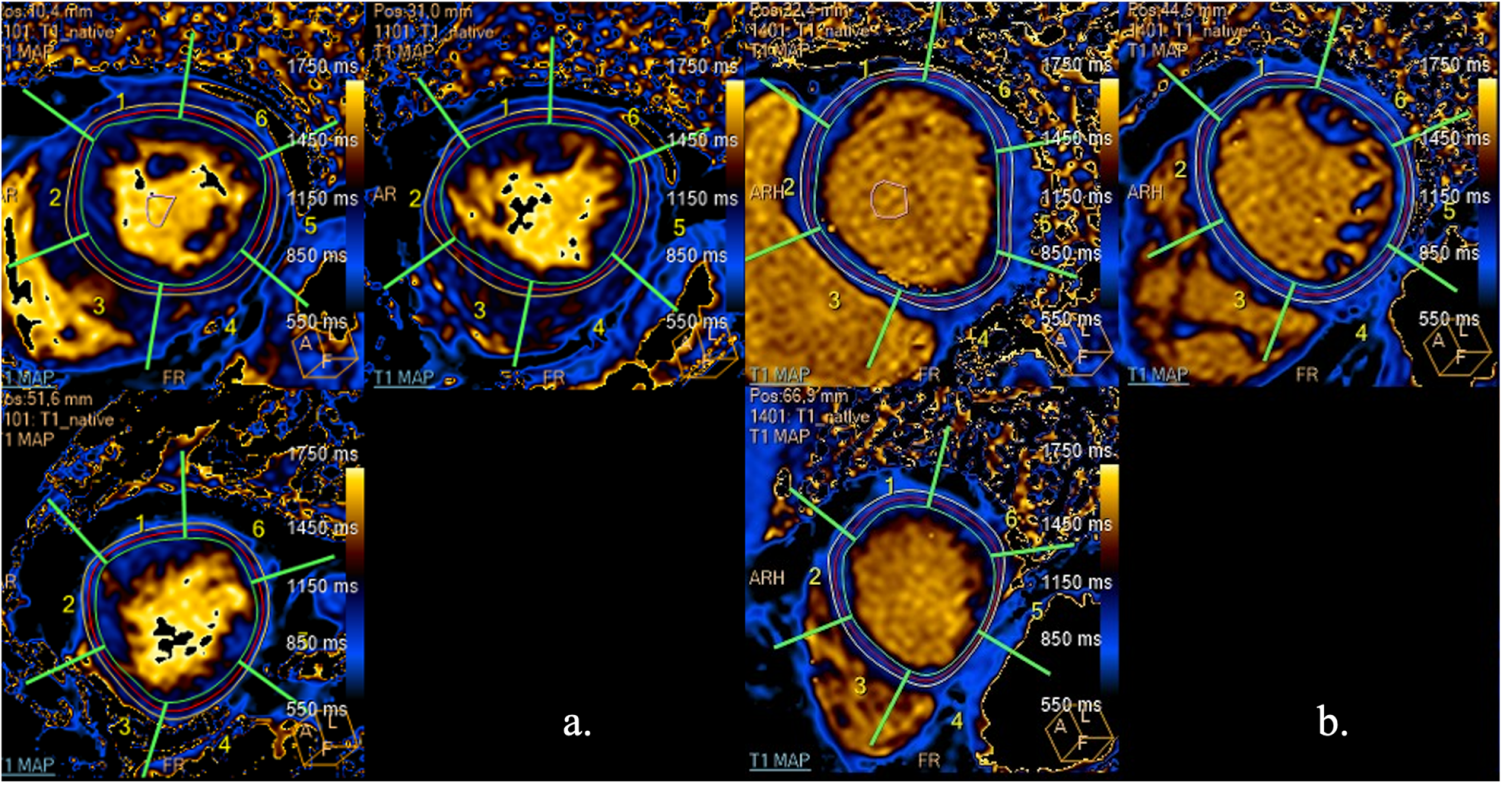
T1 mapping images performed along the cardiac short axis in a patient with cardiac amyloidosis (**a**) and Fabry disease (**b**). In comparison to reference values (i.e., 940–980 ms) at the magnetic field (1.5 T) and the sequence used (MOLLI), native T1 values result to be increased in cardiac amyloidosis (**a**) and decreased in Fabry disease (**b**)

Extracellular volume (ECV) is a quantitative biomarker of myocardial interstitial remodeling and extracellular space expansion, which can be estimated performing T1 quantification pre- and post-contrast medium administration (usually after > 10 min) and correcting for patient’s hematocrit level. Nonetheless, unlike both pre- and post-contrast T1 mapping, ECV is considered more reproducible and less affected by different acquisition techniques than native T1 mapping [86]. Extracellular space expansion is a robust indicator of myocardial fibrosis [87, 88] (Fig. 12).

## Conclusion

A knowledge of the complex phenomena that regulate T1 signal on MRI seems to be essential in clinical practice for a more effective characterization of pathological processes. We propose the following MRI protocol for high-signal lesions in basal T1 images, which we have found to be effective. (Fig. XV Supplementary Materials).

First, a T1 weighted fat-sat scan is indicated in order to distinguish between fat-containing lesions and lesions containing other substances that cause T1 shortening (e.g proteins and methemoglobin). T1 fat-sat images can also improve the detection of a hyperintense lesion deploing the rescaling phenomenon. In addition, T1 weighted fat-sat sequence is mandatory before injection of Gadolinium to avoid misinterpretation due to rescaling.

Then, we acquire a GE T1 IP/OP sequence to detect coexistence of fat and water into the lesion.

Finally, in presence of a non fat-containing hyperintense lesion, it is useful to perform a susceptibiliy-sensitive sequence, usually a GE T2* or its evolution SWI. In this way, it is possible to distinguish between hyperintensity due to presence of proteins from hyperintense lesions containg paramagnetic substances (e.g., methemoglobin, melanin, and so on).

### Supplementary information

ELECTRONIC SUPPLEMENTARY MATERIAL

## Data Availability

MRI images were extrapolated from MRI investigations performed in our hospital at General Radiology and Neuroradiology Units. They have not been previously published.

## Abbreviations

GE: Gradient echo
MRI: Magnetic resonance imaging
PD: Proton density
SE: Spin echo
