# Supplementary material for: T1 relaxation: Chemo-physical fundamentals of magnetic resonance imaging and clinical applications
Source: Insights Imaging. 2024 Aug 9;15:200. doi: 10.1186/s13244-024-01744-2 (PMC11315875; doi:10.1186/s13244-024-01744-2)

T1 relaxation. Chemo-physical fundamentals of Magnetic Resonance Imaging and clinical applications.
ELECTRONIC SUPPLEMENTARY MATERIAL

Fig.I Supplementary Materials:
Goldilocks' rule.
Protons process at a constant rate according to the Larmor's law.
Release of protons energy to molecules that tumble too slow or too fast is difficult and consequently T1 is long. On the other hand, when tumbling of molecules is similar to the precession rate of protons there is a rapid passage of energy and T1 is short.

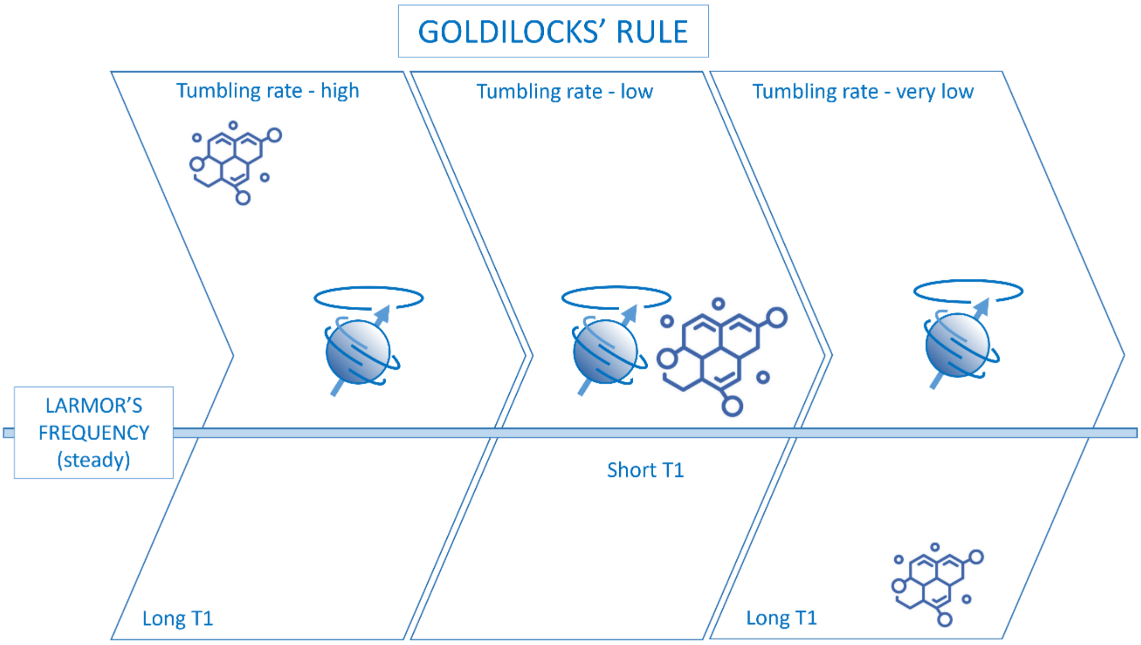

Fig.II Supplementary Materials:
Rescaling explanation.
Maintaining the same grey scale range on T1-weighted fat-sat scan, there is a shift of the lesion's signal that occupies the segment of range left empty by fat.

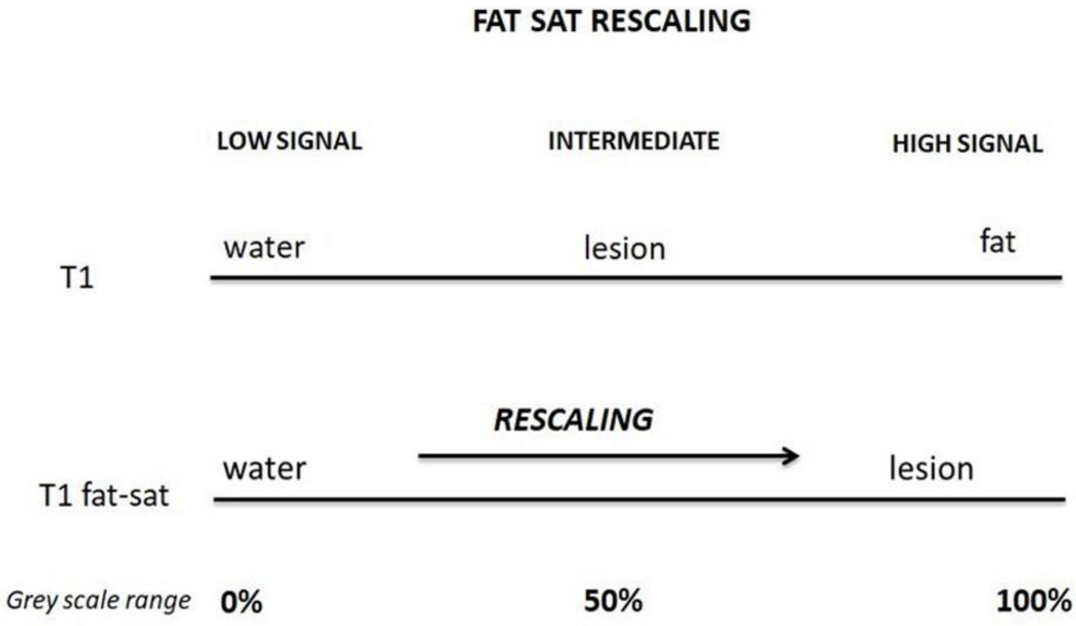

Fig.III Supplementary Materials:

Cystic mucinous adenocarcinoma of the left ovary [black arrow in (a)] with high concentrated glycoproteins (mucus) content is hyperintense on FSE T1-weighted image (a). On FSE T1-weighted fat-sat image (b) hyperintensity is much more evident due to rescaling [asterisk]. On FSE T1-weighted fat-sat Gadolinium-enhanced scan (c), the solid carcinomatous exophytic nodule enhances [red arrow]. Only on subtracted T1-weighted image (d), the thickened cyst wall enhancement can be detected [arrowheads].

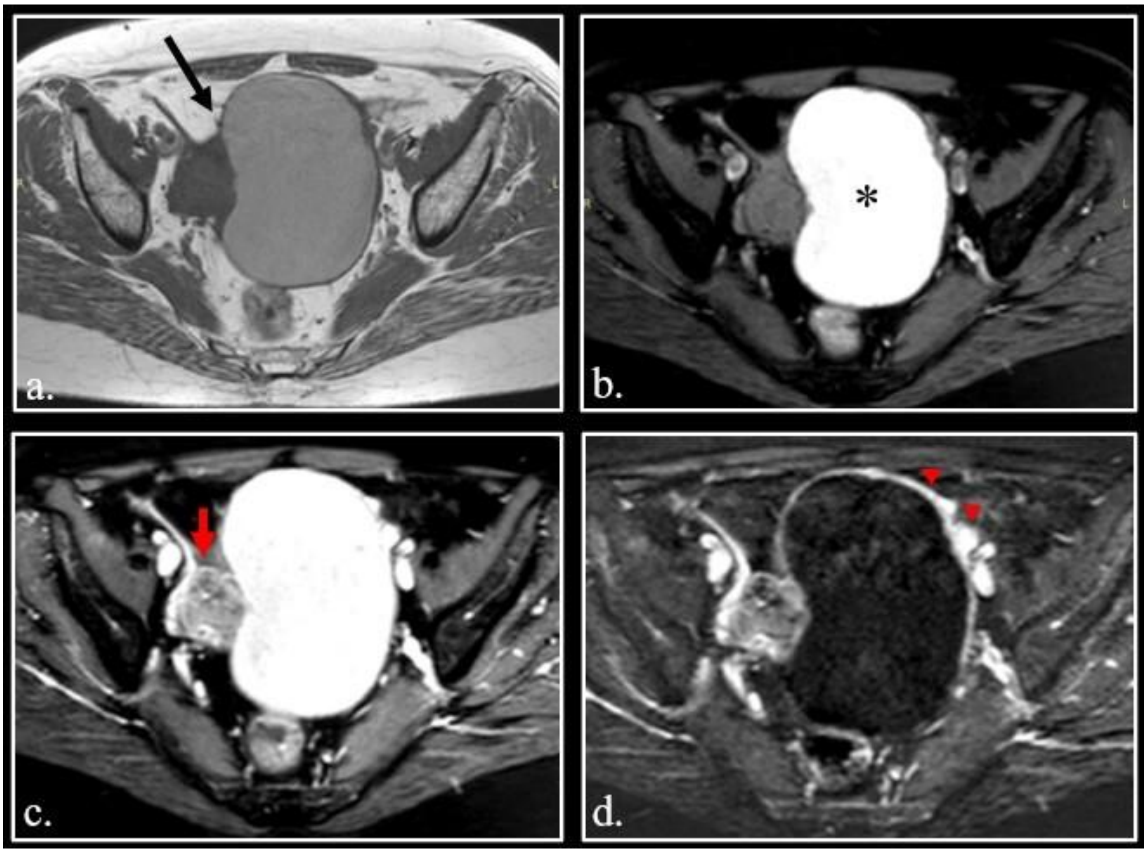

Fig.IV Supplementary Materials:
Axial T1-weighted fat-sat image (a) shows the hyperintensity [black asterisk] of a Mullerian cyst of the vagina [arrow].
Shading sign [orange asterisk] is visible on T2-weighted image (b).

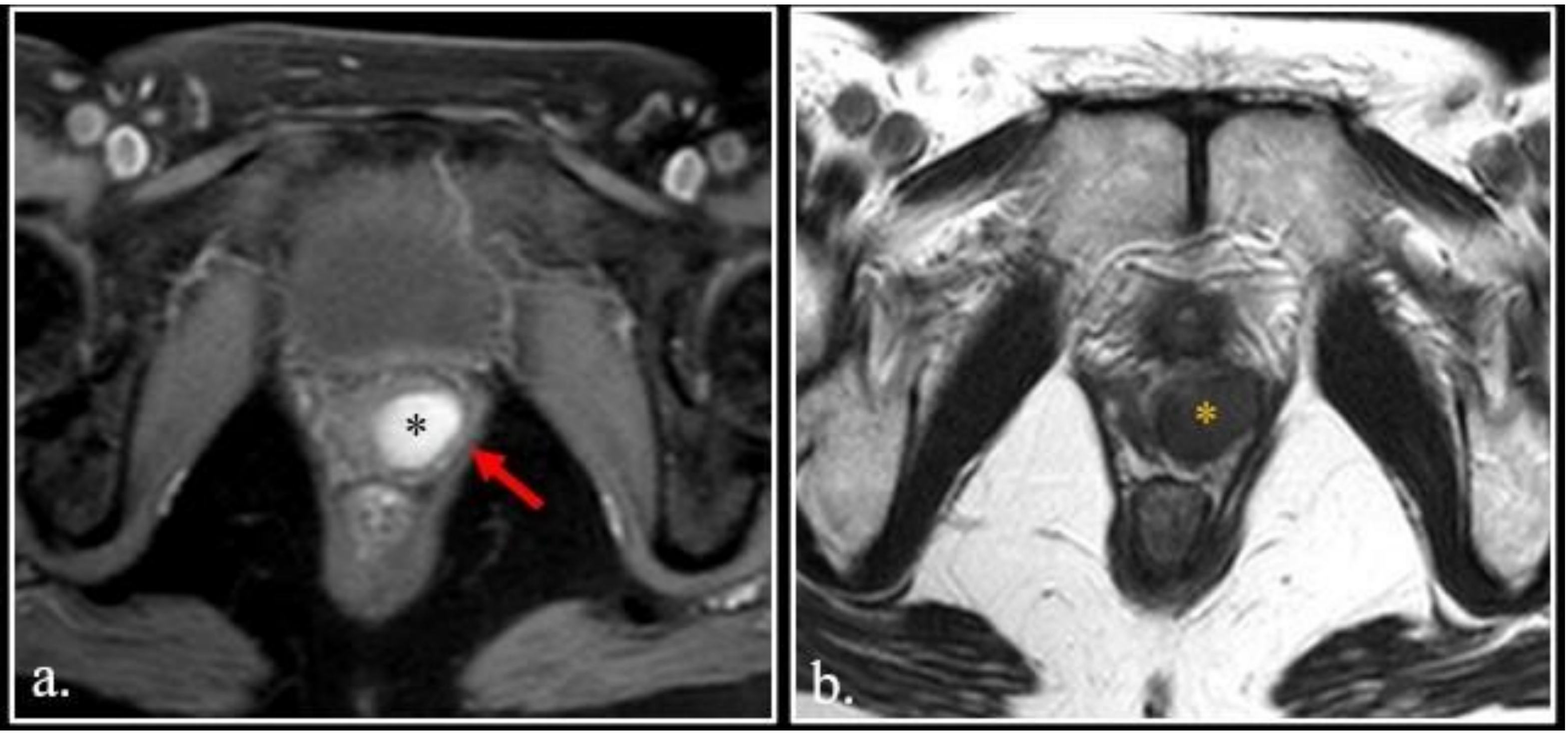

Fig.V Supplementary Materials:
In a female patient with left renal agenesis, a retrovescical hyperintense cystic lesion [arrow] from mesonephric (Wolffian) duct remnant can be seen on T1- weighted image (a). The shading sign [asterisk] is visible on T2-weighted image (b).

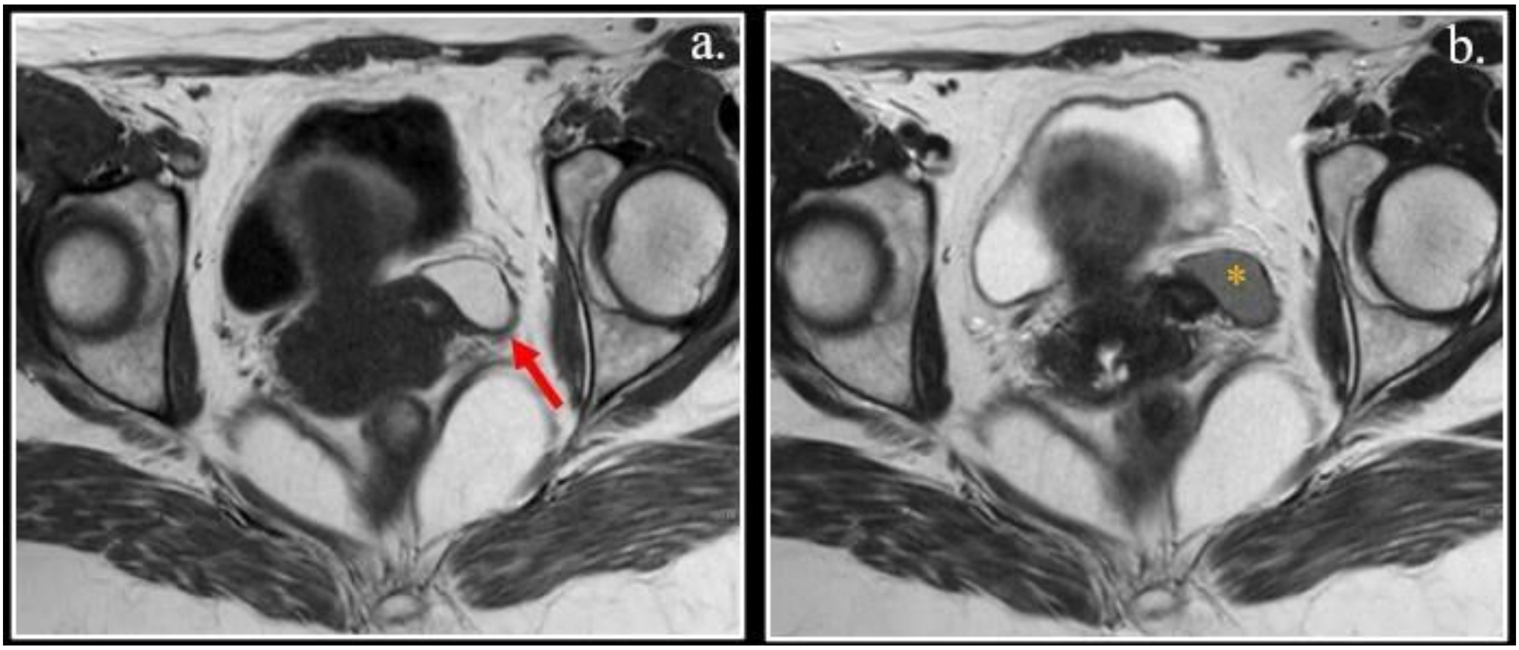

Insights Imaging (2024) Gaeta M, Galletta K, Cavallaro M, et al.

Fig.VI Supplementary Materials:

Left parietal brain cavernous angioma [arrow] is hyperintense on T1-weighted image (a) due to bleeding producing a subacute hematoma. Incomplete shading sign [red asterisk] is visible on T2-weighted image (b). SWI scan (c) demonstrates the presence of high concentration of paramagnetic substances (methemoglobin) in the walls [arrowheads] and in the lower part of the hematoma [yellow asterisk].

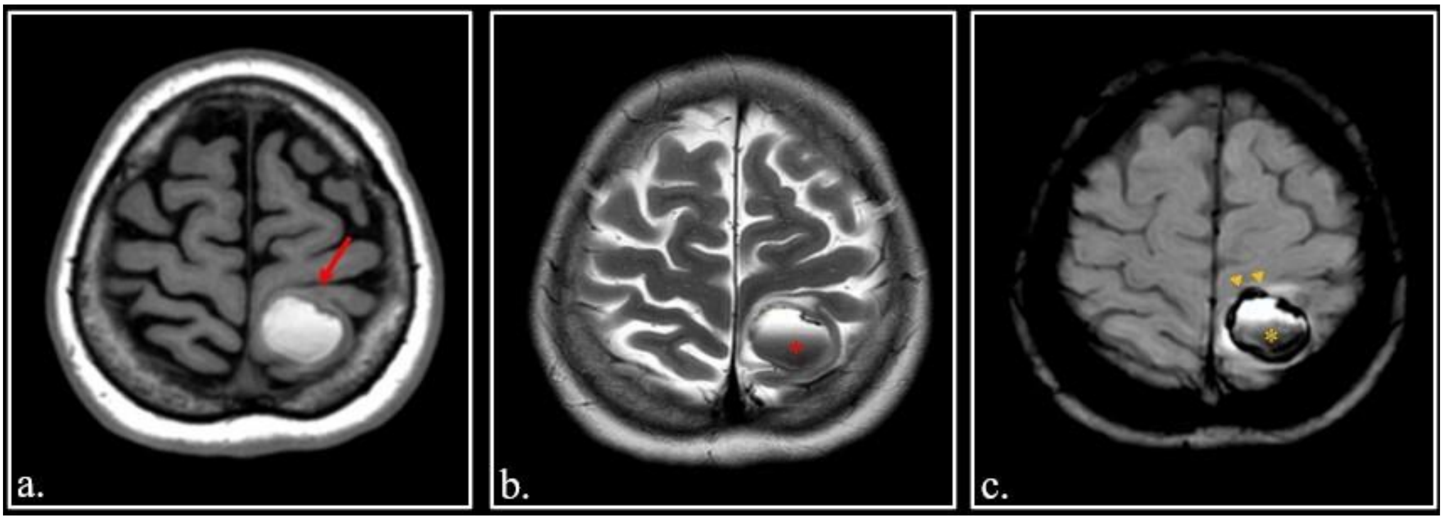

Fig.VII Supplementary Materials:

Axial FSE T1-weighted fat-sat image (a) shows an hyperintense endometrioma [arrow] of the left ovary. Note the shading sign [white asterisk] on FSE T2-weigthed image (b). On diffusion b0 scan (c), the lesion is also hypointense with consequent appearance of "T2 blackout" effect on ADC map (d).

Uterine adenomyosis [arrowheads] can also be seen (b).

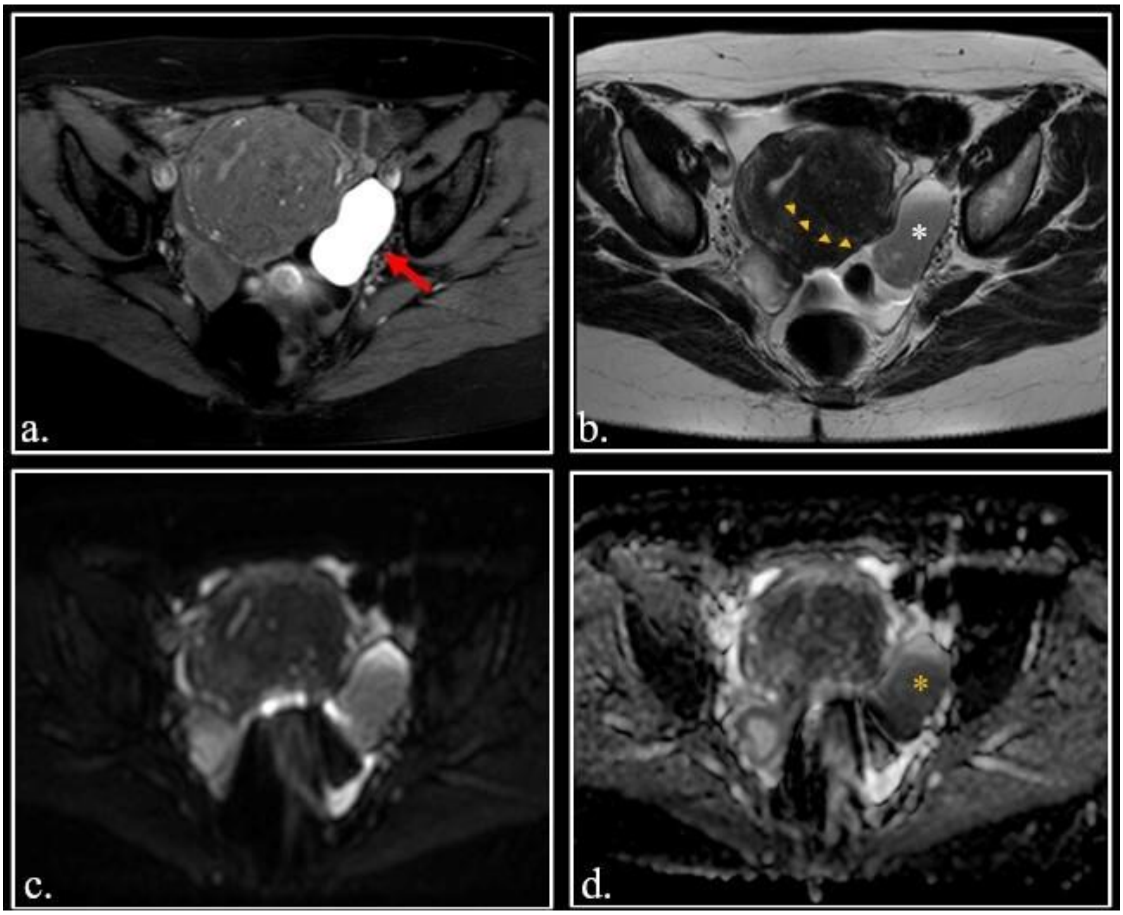

Fig.VIII Supplementary Materials:

Corpus callosum hematoma [arrow] at 24 hours.

Intracellular deoxyhemoglobin is demonstrated on SWI image (a). On T1-weighted image (b) the hematoma is isointense to brain cortex.

On a SWI (c) and T1-weighted (d) images, obtained at the 14th day, the center of hematoma is hyperintense due to presence of methemoglobin.

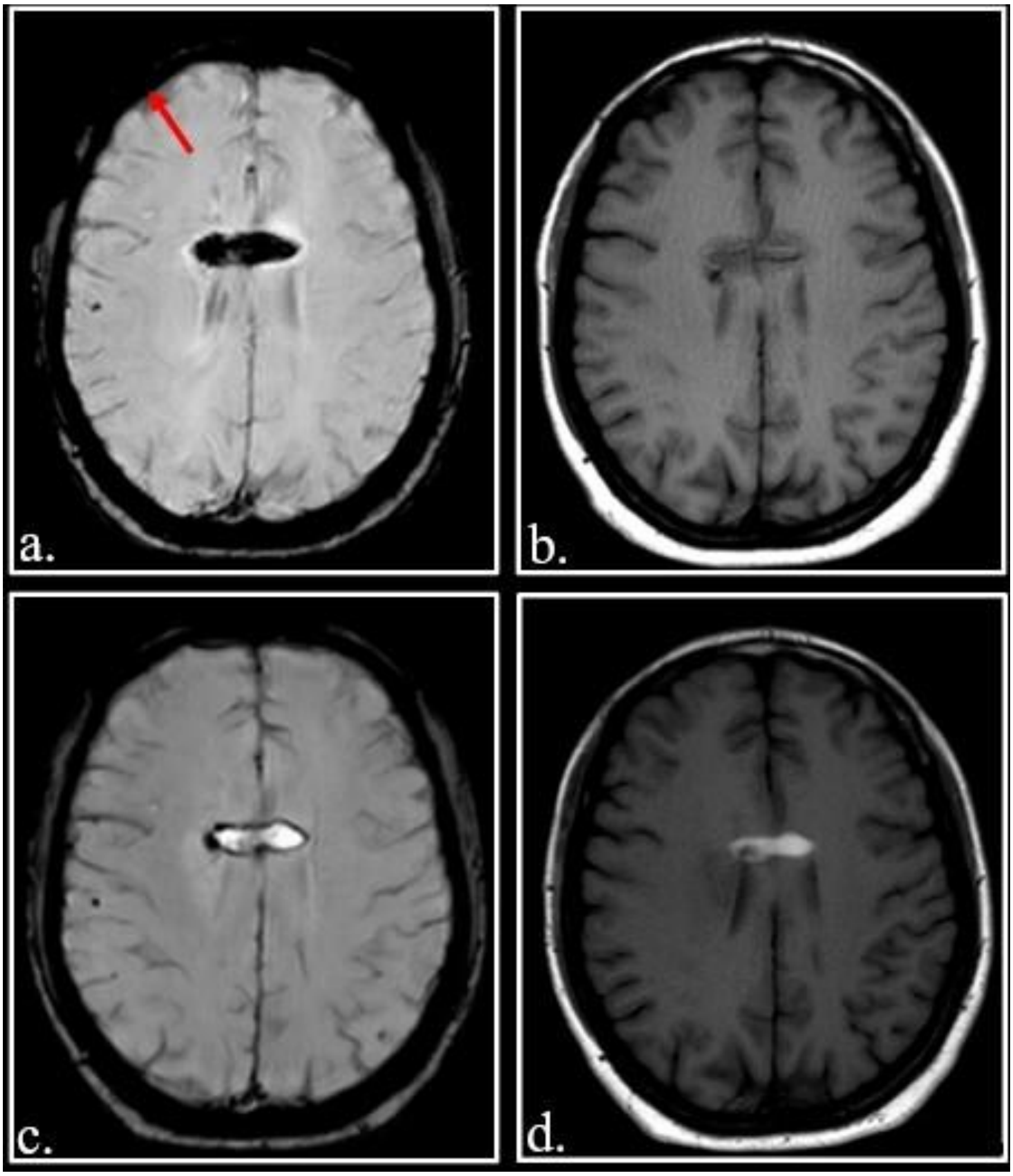

Fig.IX Supplementary Materials:

Axial FSE T1-weighted image (a) in a 32-year-old female patient with hyperintense, bilateral endometriomas [arrows] and uterine adenomyosis [arrowheads].

On T2-weighted image (b), the shading sign can be detected in some endometriomas [asterisks]. On SWI sequence (c), some cysts appear hyperintense as on T1-weighted-images, demonstrating that shortening of T1 due mainly to high protein content. On the other hand, some small cysts and myometrial cysts shows susceptibility phenomena, probably as result of higher concentration of hemosiderin [arrowheads].

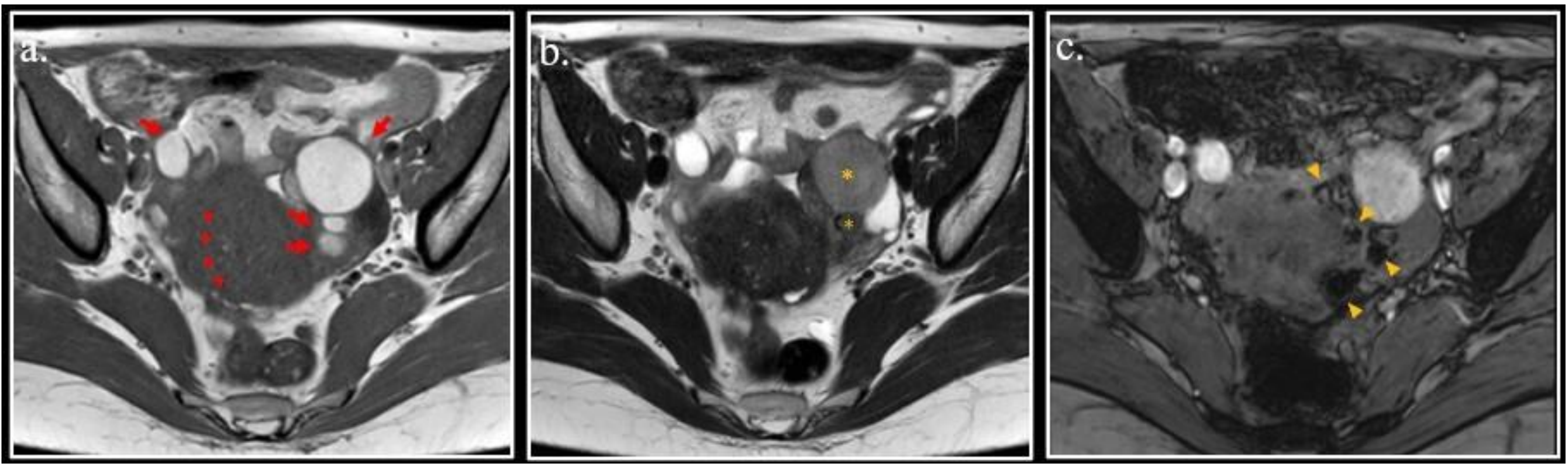

Fig.X Supplementary Materials :
Left Sylvian fissure dermoid cyst [arrows].
The lesion is hyperintense both on T1-weighted (a) as well as on a balanced Steady-State scan (b).

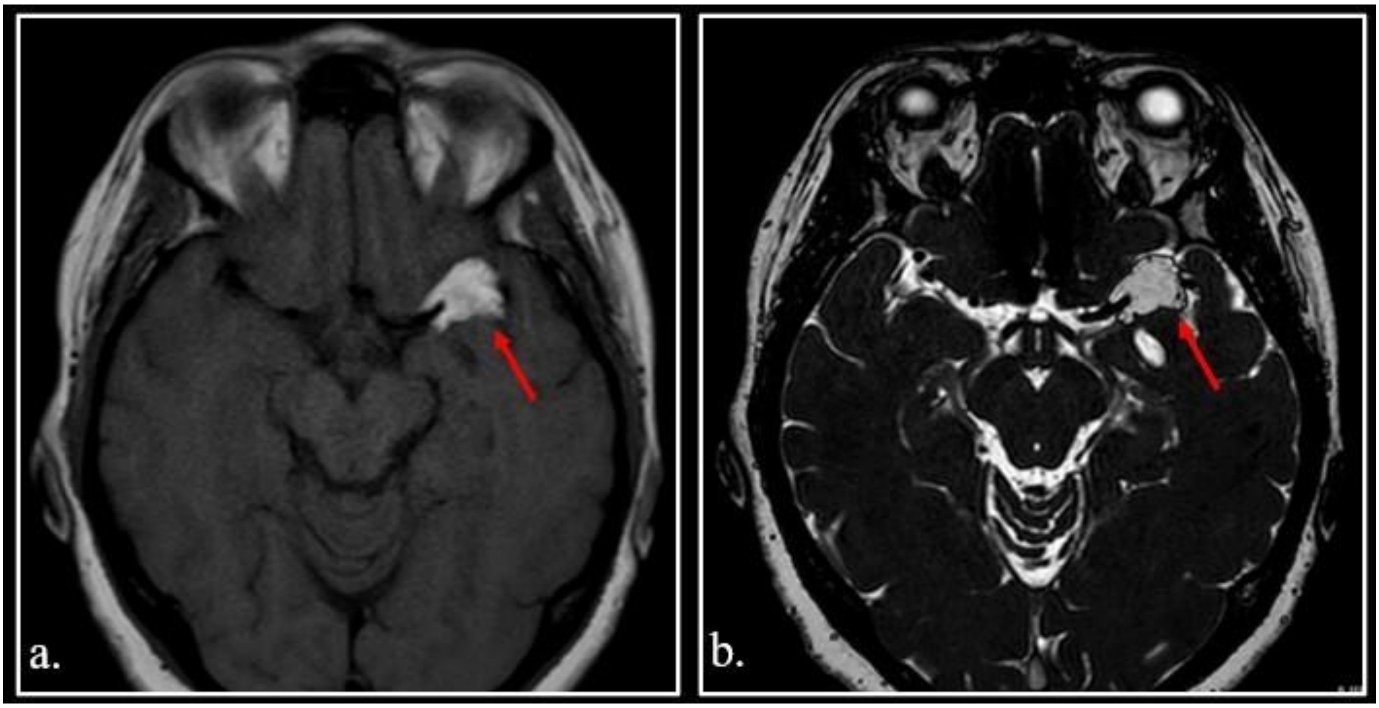

Fig.XI Supplementary Materials:

Balanced Steady-State images obtained before (a) and after Gadolinium administration (b) in a patient with a meningioma of the left posterior fossa [arrows]. Due to T1 weighting of the sequence, enhancement of the meningioma can be seen with better demonstration of the relationship between the lesion and the inferior vestibular nerve [arrowheads].

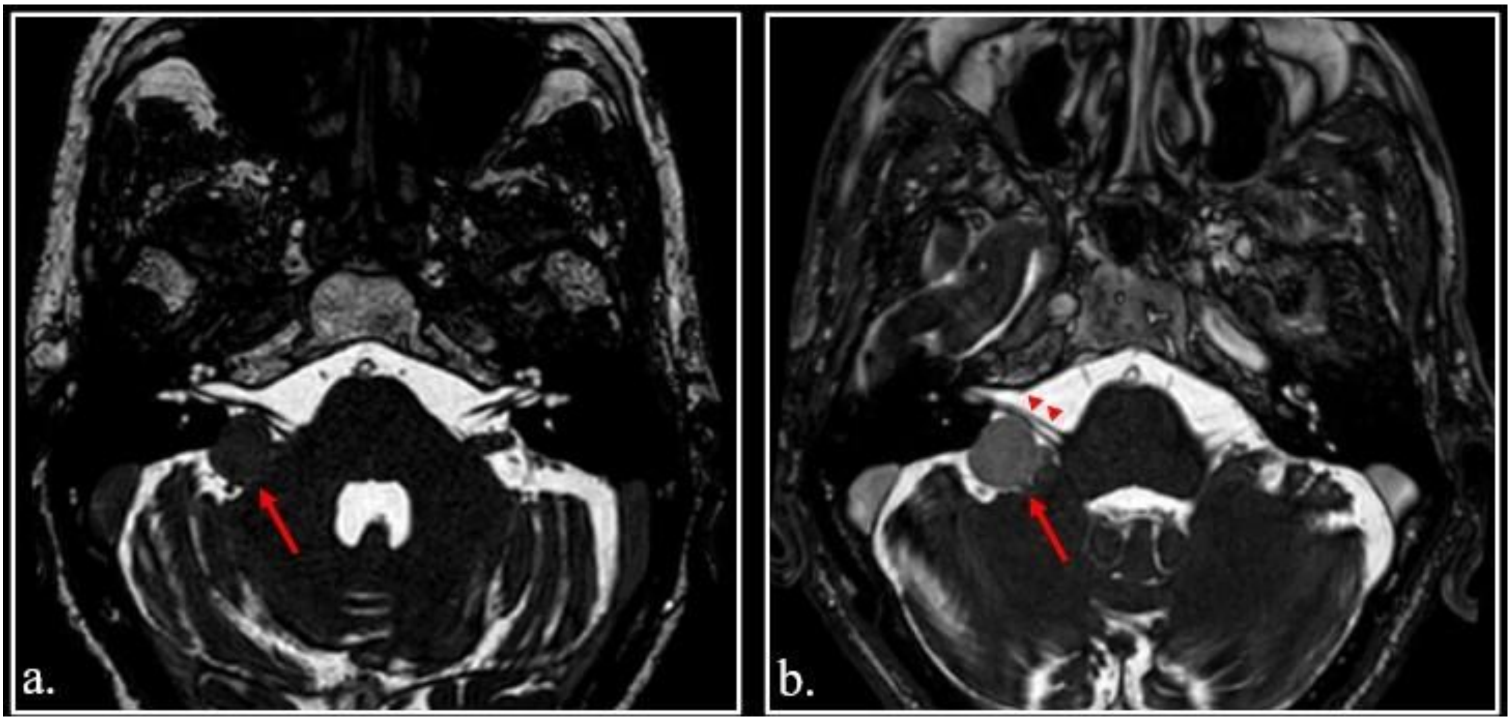

Fig.XII Supplementary Materials:

Both unenhanced FLAIR (a) and T1-weighted contrast-enhanced sequence (b) do not show meningeal abnormalities. Leptomeningeal enhancement, due to bacterial meningitis, can be detected on contrast-enhanced FLAIR (c) because the presence of small quantities of Gadolinium [arrowheads,] not detectable on T1-weighted sequence, is sufficient to shorten T1 and prevent CSF suppression.

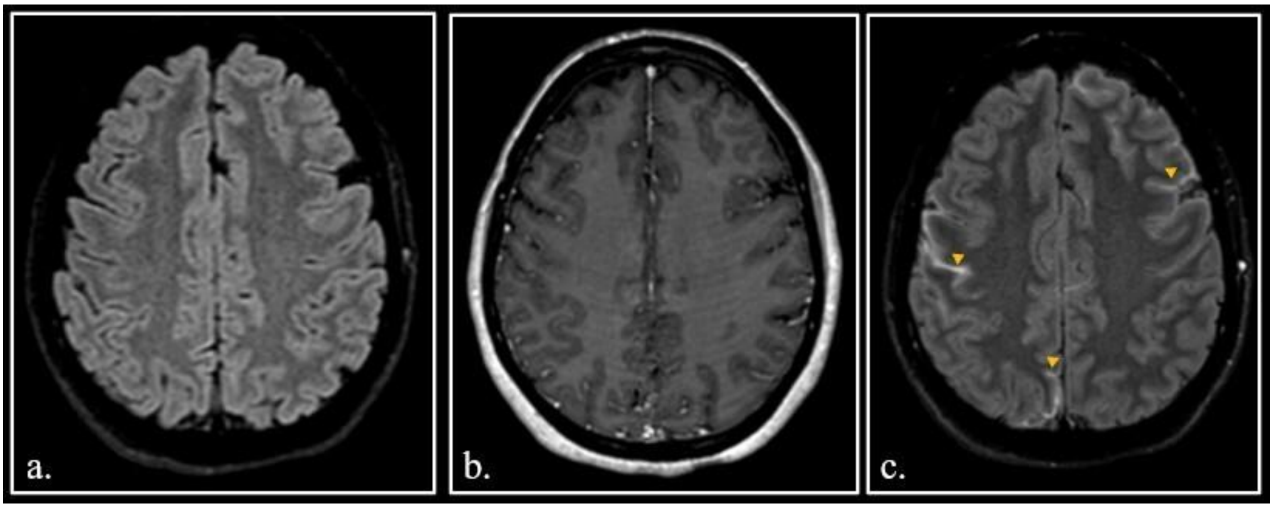

Fig.XIII Supplementary Materials:
Signal of a demyelinating plaque [arrowheads] of the cervical cord is more intense in STIR (a) than in FSE T2-weighted scan (b).

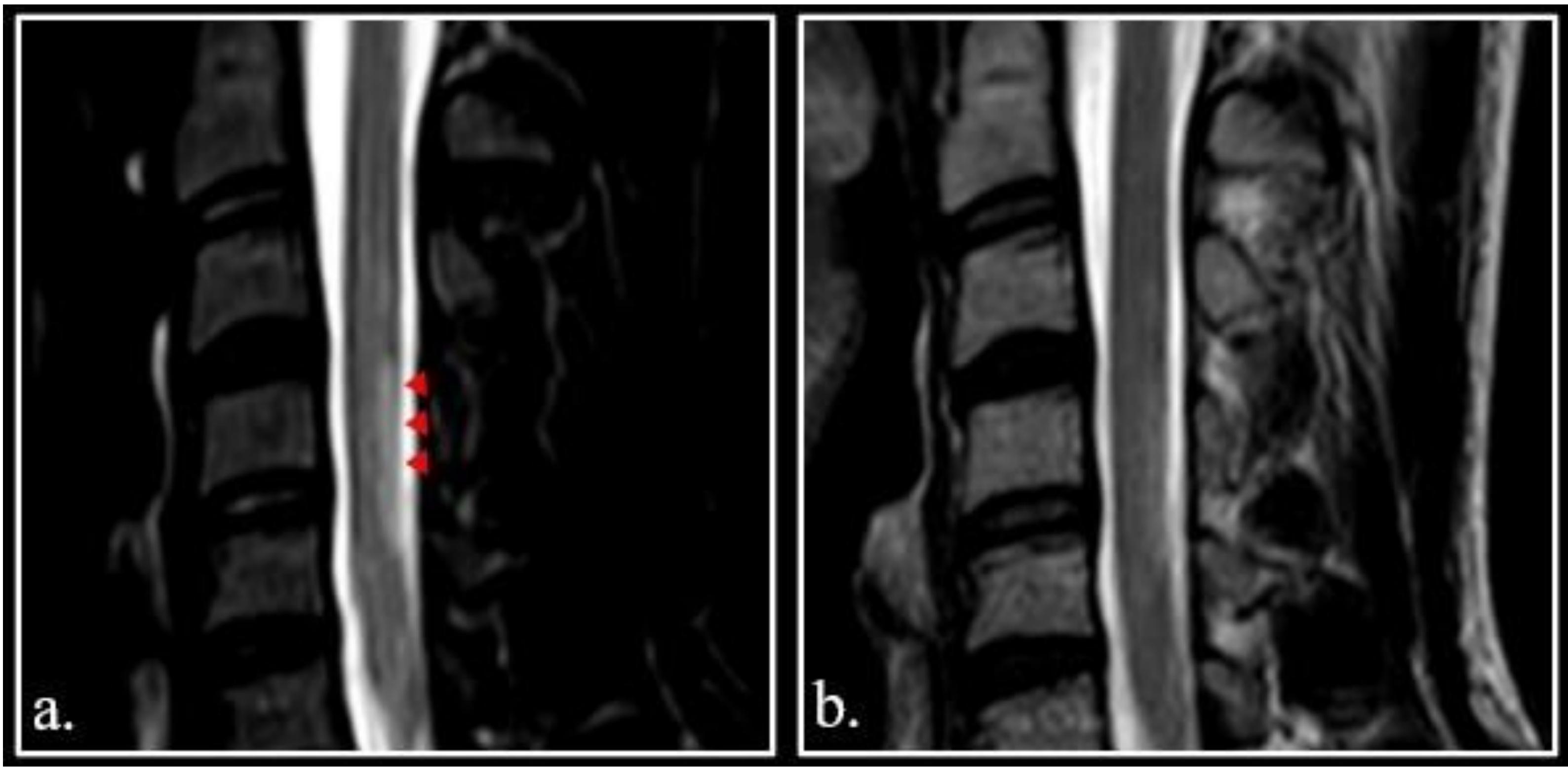

Fig.XIV Supplementary Materials:
Right ovary endometrioma [arrow] is hyperintense on unenhanced FSE T1-weighted image (a).
On STIR scan (b), there is a complete loss of signal [red asterisk] mimicking a fat content.
FSE T1-weighted fat-sat image (c) demonstrates that the cyst does not contain fat [black asterisk].

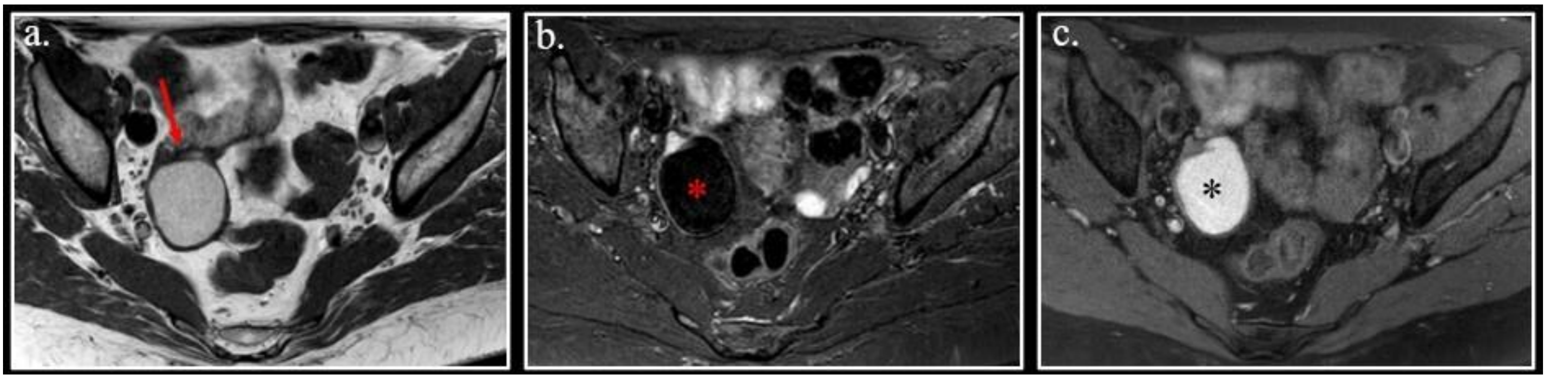

Fig.XV Supplementary Materials:
Suggested MRI protocol for T1 hyperintense lesions.

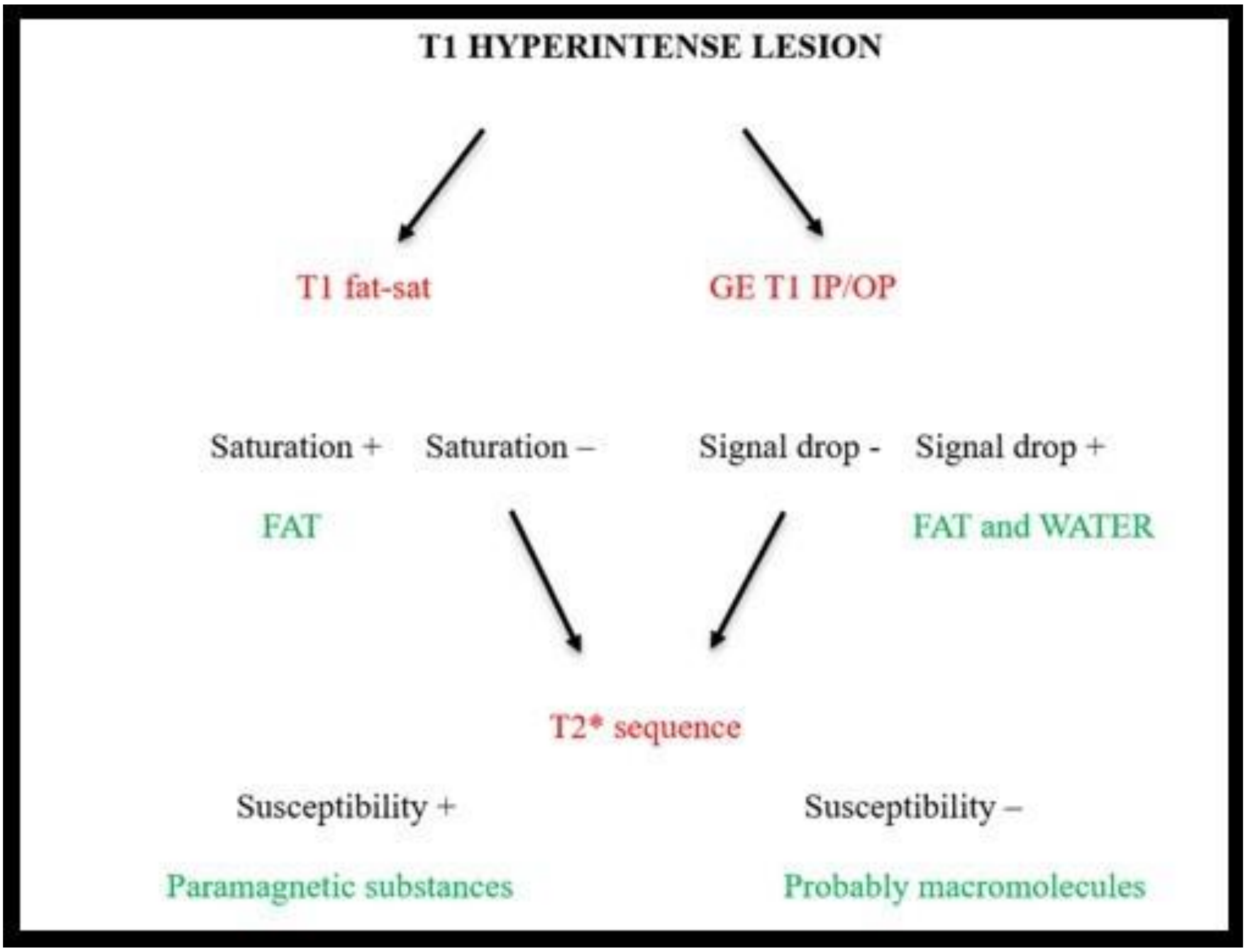

Supplement: Supplementary file 1 — ELECTRONIC SUPPLEMENTARY MATERIAL [file 13244_2024_1744_MOESM1_ESM.pdf]
